# The Role of Urban Environments in Promoting Active and Healthy Aging: A Systematic Scoping Review of Citizen Science Approaches

**DOI:** 10.1007/s11524-022-00622-w

**Published:** 2022-05-19

**Authors:** G. E. R. Wood, J. Pykett, P. Daw, S. Agyapong-Badu, A. Banchoff, A. C. King, A. Stathi

**Affiliations:** 1 School of Sport, Exercise and Rehabilitation Sciences, University of Birmingham, Birmingham, UK; 2 School of Geography, Earth and Environmental Sciences, University of Birmingham, Birmingham, UK; 3 Department of Epidemiology and Population Health, Stanford University School of Medicine, Stanford, CA USA; 4 Department of Medicine, Stanford Prevention Research Center, Stanford University School of Medicine, Stanford, CA USA

## Abstract

Promoting active and healthy aging in urban spaces requires environments with diverse, age-friendly characteristics. This scoping review investigated the associations between urban characteristics and active and healthy aging as identified by citizen science (CS) and other participatory approaches. Using a systematic scoping review procedure, 23 articles employing a CS or participatory approach (participant age range: 54–98 years) were reviewed. An inductive and deductive thematic analysis was completed to (a) identify local urban barriers and facilitators and (b) map them against the World Health Organization (WHO) Checklist of Essential Features of Age-Friendly Cities. A new Citizen Science Appraisal Tool (CSAT) was developed to evaluate the quality of CS and other participatory approaches included in the reviewed articles. A range of interconnected urban barriers and facilitators was generated by residents across the personal (e.g. *perceived safety*), environmental (e.g. *unmaintained infrastructure*), socio-cultural (e.g. *cross-cultural activities*), economic (e.g. *affordable housing*) and political (e.g. *governmental support to migrant communities*) domains. Mapping the barriers and facilitators to the WHO age-friendly checklist underscored the checklist’s relevance and elucidated the need to explore *barriers for migrant and cross-cultural communities* and *neighborhood development and alterations*. The CSAT demonstrated strengths related to active engagement of residents and study outcomes leading to real-world implications. To advance the potential of CS to enrich our understanding of age-friendly environments, employing co-production to enhance relevance and sustainability of outcomes is an important strategy. Overall, employing CS highlighted the value of systematically capturing the experiences of older adults within studies aimed at promoting active and healthy aging.

**Supplementary Information:**

The online version contains supplementary material available at 10.1007/s11524-022-00622-w.

## Introduction

The combination of increasing levels of urbanisation and an aging population worldwide has highlighted the importance of city-level public health initiatives targeting older adults [1, 2]. Urban environments and their physical and socio-ecological characteristics can significantly impact a resident’s ability to age healthily [3, 4]. To operationalize this knowledge globally, the World Health Organization (WHO)  launched guidance in 2007 for developing age-friendly environments based on the concept of active aging [5], targeting eight urban domains: outdoor spaces and buildings, housing, transportation, respect and social inclusion, social participation, communication and information, community support and health services, and civic participation and employment. The WHO guidelines have been influential in directing the global urban planning agenda towards developing age-friendly environments. However, successful implementation of this agenda requires a comprehensive understanding of the uniqueness and complexity of urban environments and their competing social, economic and political forces [6–8].

There is a large public health literature and policy drive towards ‘age-friendliness’ and ‘aging in place’, which refers to aging at home rather than in institutional care [9]. Despite this, the concept of active aging still lacks sufficient attention to the diverse range of contextual urban socio-ecological elements required to support older adults’ health and well-being [10]. Person-specific influences, inaccessible physical environments, migration behaviors, multiple forms of deprivation and local community cohesion are just a few urban socio-ecological elements that influence a healthy and active lifestyle [11–14]. As these factors vary across communities and populations, identifying and prioritising urban characteristics that contribute to developing age-friendly environments thus remains a challenge for research and policy [15], particularly in relation to characteristics that are influential at the local level [16]. In turn, the unique pathways between urban features and healthy aging and the intersections between older people and their communities need to be more fully articulated. Understanding these at a local contextual level is important to effectively shape the opportunities to live and age in health-enhancing ways.

To address local-level variations and deliver more meaningfully on the age-friendly agenda, an increasing number of age-friendly strategies have focused on the need to foster effective engagement of older adults [17, 18]. There are multiple participatory practices for engaging members of the public in research, which overlap in their processes but differ in the level of ‘power’ given to residents [19, 20]. Across a variety of applications, citizen science (CS) can effectively engage laypeople in all stages of the research to co-produce its processes and outcomes [21, 22]. Through its systematic application in the health and urban domains, the local expertise of residents has been successfully incorporated into multi-level outcomes for altering local urban communities to promote health and well-being [23–25], which has effectively advanced the development of age-friendly environments [17].

Notwithstanding these promising studies on the potential for CS, a rigorous assessment and evaluation of CS approaches is required to understand its potential for effectively engaging individuals in shaping outcomes for their local spaces [26–28]. Previous studies advancing CS have produced tools evaluating elements of CS methods, public participation and outputs [28–31]. However, greater attention to evaluation processes needs to be paid to the diversity of approaches within such research methodologies, including the degree to which they are contributory (*for the people*), collaborative (*with the people*) or co-productive (*by the people*) [21, 32], as well as how sustainable and significant the outcomes are from the perspective of older adult community members themselves [33, 34].

Emphasising collective forms of CS to enhance age-friendly urban initiatives is especially pertinent, given what is already known about the mutually beneficial importance of social connection, walkability and physical activity in older age groups [35, 36]. This systematic scoping review aimed to investigate the urban barriers and facilitators identified through CS and other participatory approaches to be associated with active and healthy aging. The objectives of this review were to:

1. systematically review the outcomes of CS studies in which community members identify the characteristics influencing active and healthy aging in urban environments;
2. map the outcomes of the scoping review against the WHO Checklist of Essential Features of Age-Friendly Cities [37] and explore similarities and differences; and
3. develop and apply a new Citizen Science Appraisal Tool to evaluate the quality of the CS methods included in the scoping review.

## Methods

A systematic scoping review was chosen as a suitable approach to identify and map the key urban characteristics presented by CS studies and highlight knowledge gaps [38]. The protocol was guided by the PRISMA systematic review guidance [39] and scoping review extension checklists [40] and reviewed by academic experts in the fields of CS, healthy aging and political geography.

### Study Eligibility, Information Sources and Search Strategy

Studies were eligible if they met the criteria in Table 1. CS can take various forms and employ varying degrees of public participation and is an evolving approach with continuously changing terminology [41, 42]. This review aimed to be inclusive of any studies that have directly engaged older adults in one or more stages of the research process using CS or other participatory approaches [19, 43].

**Table 1:** Inclusion criteria and guidance

Criteria	Guidance
Studies engage residents aged 60 years or above	Residents must be aged 60 or above, or if other age groups are engaged, then residents must have an average age of 60 years
Studies are completed in an urban setting	Studies include any of the following keywords to represent urban settings: (1) urban (environment, area, setting), (2) built (environment, area, setting), (3) age-friendly (city, cities, environments), (4) city/cities, (5) physical (environment, area, setting), (6) outdoor (environment, area, setting), (7) inclusive community/ies and (8) neighborhood/s. Studies that also undertake a comparison between urban and rural settings are included if there is a clear separation of results and outcomes for both settings
Studies must employ a citizen science or participatory approach in any or all stages	Citizen science is defined as resident-engaged citizen science [17] in which laypersons, in particular older adults, engage or participate in research with the intention of scientific advancement [21, 43]. Studies that use the term ‘citizen science’ or directly engage older adults in any or all stages of the research using a participatory approach can be included
Studies aim to investigate the relationship between urban environments and active and healthy aging (or a component of active aging)	Active aging is defined as "the process of optimizing opportunities for health, participation and security in order to enhance the quality of life as people age" [44]. Studies include any of the following keywords: (1) active aging, (2) healthy aging, (3) successful aging, (4) aging well, (5) positive aging, (6) productive aging, (7) meaningful aging, (8) active lifestyle and (9) healthy lifestyle. Studies that have used any of active aging keywords above and explored the following domains will also be included to incorporate the ‘active’ element [46]: (1) physical activities, (2) social activities, (3) economic activities, (4) cultural activities, (5) spiritual activities, (6) civic affairs and (7) labour force activities

An iterative approach [10, 46] allowed the search strategy and keywords to be reviewed and revised based on the relevant literature identified throughout the review process. A preliminary trial search of databases was completed (GW) and discussed with 2 reviewers (AS, JP) to produce a refined set of keywords. The keywords combined all possible combinations, English and American spelling, Boolean operators and truncation (see Supplementary Material 1). Searches were completed on MEDLINE, Embase, PsycINFO, Social Policy and Practice, CINAHL, PubMed, Scopus and Cochrane Library between 01/03/2020 and 01/06/2020, with an updated search completed between 01/06/2020 and 19/06/2021. Articles were recorded using EndNote (X9, 2019) reference management software, and duplicates, non-English language and grey literature were excluded.

### Screening and Data Extraction

**Fig. 1:**
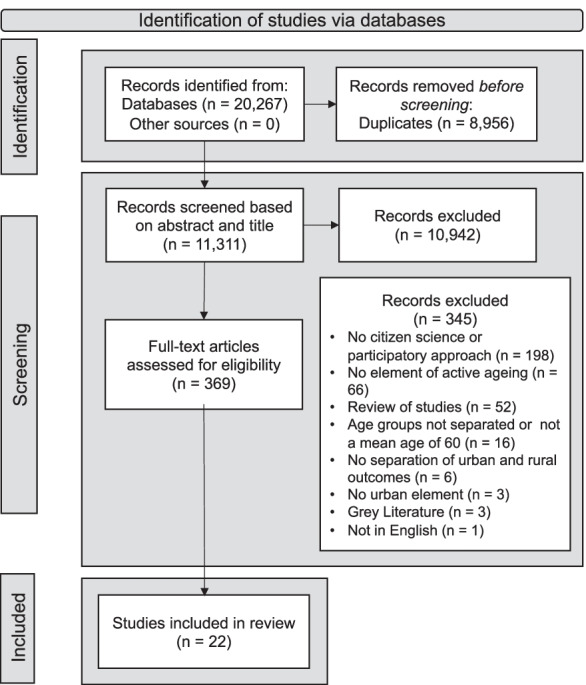
Flow diagram of article selection. Adapted from PRISMA flow diagram 2021 [39]

Titles and abstracts were screened by the lead reviewer (GW), with a randomly selected 20% of articles independently screened by two reviewers (PD, SA). Articles that did not meet inclusion criteria were excluded. The same process was completed for full-text screening, with a fourth reviewer (AS) present to discuss discrepancies (Fig. 1). Data were extracted from the final set of included studies (GW) and independently checked (PD). The data extracted included (1) study aims, setting, design and method; (2) focus of the study (e.g. active aging focus, urban focus); (3) participant characteristics; (4) details of CS or other participatory approaches; (4) urban environment characteristics or methodology outcomes; and (5) conclusions and recommendations.

### Citizen Science Appraisal Tool

The Citizen Science Appraisal Tool (CSAT) (Table 3) was developed to evaluate the quality of CS and other participatory approaches. Quality is defined here in relation to the levels of active engagement and partnerships utilised by CS studies [47]. These two factors of engagement and partnerships are evaluated through a lifecycle approach [48] starting with the aims of a study through to its outcomes and future impacts and consider the scientific standards, participation, data quality and dissemination, which are elements of good quality CS [22, 49]. The tool development was guided by the European Citizen Science Association (ECSA) 10 principles [22] and Critical Appraisal Skills Programme (CASP) tools [50, 51] and encompassed three levels of engagement: contributory (*for the people*), collaborative (*with the people*) and co-productive (*by the people*) [21] (see Supplementary Material 1).

The tool gives equal weight to all questions to encompass both CS engagement and scientific standards. Active engagement and developing real-world outcomes are crucial elements of CS [47, 52] alongside demonstrating validity, transparency and appropriateness of methods and data [53–55]. Providing equal weight enables the tool to assess the quality of CS engagement and scientific standards, which are elements of good quality CS [22, 49]. All included studies were evaluated using the CSAT (GW), with 20% of evaluations independently checked (AS, JP).

### Data Charting and Analysis

A descriptive summary of the 23 included studies was completed, followed by a thematic analysis and narrative synthesis. This was deemed suitable to systematically identify, map and synthesise the qualitative nature of the methods and outcomes. An inductive thematic analysis [56, 57] was completed to generate community-identified local urban barriers and facilitators of active and healthy aging. The lead author (GW) completed initial coding, with codes, subthemes and main themes discussed with two reviewers (PD, SA). A second stage of coding was completed (GW) and discussed with three reviewers (PD, AS, JP). The final themes were confirmed by all reviewers. Following this process, two articles [58, 59] were excluded from this analysis as their outcomes focused on an overview of their methodology rather than urban characteristics.

A deductive thematic analysis [56, 57] was completed to map the barrier and facilitator themes against the WHO Checklist of Essential Features of Age-Friendly Cities [37]. This checklist presents 8 age-friendly features that encompass 84 relevant urban descriptors (https://www.who.int/ageing/publications/Age_friendly_cities_checklist.pdf). The lead author (GW) completed initial coding which was discussed with two reviewers (PD, SA). A second stage of coding was completed (GW) and discussed with three reviewers (PD, AS, JP) in which themes mapped against the WHO descriptors were finalised. Lastly, a narrative synthesis [60, 61] was completed to provide a summary of evidence on the CS methods and their quality and potential capacity to strengthen age-friendly urban initiatives.

## Results

Twenty-three studies were reviewed (Table 2), with the quality of CS approaches evaluated using the CSAT (Table 3). Most studies were published between 2015 and 2020 (*n* = 19) and took place in the UK (*n* = 6), Canada (*n* = 6) and/or USA (*n* = 4), Australia (*n* = 2), Hong Kong (*n* = 2), Netherlands (*n* = 1), Sweden (*n* = 1), New Zealand (*n* = 1) and Finland (*n* = 1). Data collection duration ranged from 2 weeks (*n* = 2), 1 to 12 months (*n* = 12), 3 years (*n* = 1) and during spring or fall (*n* = 2). Duration was not reported by 7 studies. The studies included observational (*n* = 20) and cross-sectional (*n* = 3) research designs. Residents were aged between 54 and 98, with one study not reporting the age of participants [62]. Ethnicity, sex and other demographic variables are presented in Table 2.

**Table 2:** Summary and characteristics of the twenty included studies, in order from CSAT highest to lowest quality

Author, year, location	Study aim, duration, urban setting & active aging element	Population	Citizen science/participatory research design & methods	Main findings
Barrie et al. (2019), Australia [82]	**Aim**: to test a new smart phone–based audit tool using an innovative methodology—citizen science—in order to explore how and why older people engage with public green spaces **Duration**: 6 to 10 weeks of data collection with focus group after **Urban setting**: public green spaces (i.e. parks, gardens) **Active aging element**: aging well	***N*** = 15 (female = 12, male = 3) **Age range** = 60–84 years **Ethnicity** = not reported **Target population** = not reported	**Design**: citizen science approach and co-created model **Methods**: Audit of green spaces using digital audit tool, interviews & co-analysis	**1) Key design elements** of public green spaces included seating, street trees, natural bushland, park trees and water (creeks, lakes, rivers) **2) Main reasons for visiting green spaces** included “On my way to somewhere else”, “to relax”, “to exercise” and “to meet others”. The majority of citizen scientists spent “Less than 15 min” in green spaces followed by “15 to 30 min”
Gustafsson et al., (2018), Sweden [63]	**Aim**: to describe life filming as a means of participatory approach in relation to older community-dwelling persons and the design of their local environment **Duration**: took place in the fall **Urban setting**: 1 of 10 urban districts in the city of Gothenburg **Active aging element**: healthy aging, active aging	***N*** = 7 (female = 5; male = 2) **Age range** = 69–78 years **Ethnicity** = not reported **Target population** = community-dwelling persons aged 65 years or older, including individuals with visual impairments	**Design**: participatory approach **Methods**: life filming & group meetings	**5 themes for central aspects of life filming**: 1) Anchoring the concept of participation 2) Practical application of life filming 3) The film as a product 4) Making a real difference 5) An identity as a capable older person **Life films highlighted the barriers of the environment as** hazardous traffic situations, lack of or incorrect weather protection at tram stations, difficulties handling the digitalized society, barriers to a wheelchair user accessing buildings and thoughts about future housing opportunities
von Faber et al. (2020), Netherlands [64]	**Aim**: to describe how participatory video design can add knowledge about the preferences and needs of older people about the improvement and preservation of their local environment **Duration**: 1-week data collection, 1-week workshop **Urban setting**: 2 cities, the Hague and the city of Leiden **Active aging element**: active aging through participation in research	***The Hague*** ***N*** = 21 (female = 14, male = 7) **Age range** = 63–90 **Ethnicity** = Dutch, Chinese Surinamese **Target population** = residents over 60 years or older ***The city of Leiden*** ***N*** = 9 (female = 3, male = 6) **Age range** = not reported **Ethnicity** = not reported **Target population** = residents over 60 years or older	**Design**: participatory research **Methods**: participatory video design and workshop	**1) Topics of the films** included outdoor spaces, housing, social participation, communication and information, and social contacts and community support **2) The process of** participation demonstrated telling a story, learning something new, forming new friendships and owning the story were strengths of the process
Tuckett et al. (2018), Australia [65]	**Aim**: (1) what are the features that help or hinder access to a seniors’ centre? (2) what are the features of the physical environment surrounding a seniors’ centre that help or hinder physical activity (walking)? (3) In what ways can older adults acting as citizen scientists bring about changes to their local environment? **Duration**: 3 months **Urban setting**: community centre adjacent to parklands **Active aging element**: active aging, successful aging	***N*** = 8 (female = 7; male = 1) **Age** = 65 years or over **Ethnicity** = not reported **Target population** = member of the Burnie Brae, aged 65 years or over, able to walk unaided, actively engaged in a range of social activities	**Design**: exploratory citizen science community-based empowerment approach **Methods**: our voice citizen science framework using a non-medical mobile application, discussion group and activated sessions	**Hindrances and facilitators to physical activity, walking and access**: 1) Parks/playground: sleeper that is at safety risks, need care to step on cement slabs, need for more covered shade over the tables 2) Footpaths: Shared pathways between bikes and pedestrians were both a facilitator and hindrance, loose gravel paths are a hindrance and damaged footpaths are hazardous footpaths 3) Traffic-related safety/parking: lack of parking, cars parked blocking drivers view, need for repainting of car park lines
Ronzi et al. (2016), UK [66]	**Aim**: to stimulate collective action and advocacy to affect policy and empowerment by (i) encouraging dialog between older people and city stakeholders at the exhibition and (ii) ensuring that older people’s views were brought to the attention of city stakeholders so that they could include their concerns in decision making and planning processes for an AFC **Duration**: 7 months **Urban setting**: city of Liverpool **Active aging element**: respect and social inclusion (element of active aging/age-friendly cities)	***N*** = 26 (female = 19; male = 7) **Age range** = 60– > 85 **Ethnicity** = White British (77%); Asian British (8%); Black British (12%); Other White Background (3%) **Target population** = older adults from 4 electric wards	**Design**: CBPR **Method**: photovoice, semi-structured interviews & focus groups	**Opportunities, challenges and solutions of using photovoice**: 1) The photoproduction process as a way to raise participants’ consciousness 2) Photographing negative aspects which included wanting to portray the environment in a positive light or not perceiving negative aspects 3) Photographing negative social concepts which participants found hard to photograph (i.e. social isolation) 4) Time period for taking photographs 5) Ethical aspects 6) Overcoming challenges such as addressing ‘missing photographs’ and photography training **Barriers and facilitators to respect and social**: 1) Bus station considered very uncomfortable due to a lack of protection from the wind and an enclosed bus and train station would be an enabler 2) Public toilets that are not accessible or inviting are a barrier to social inclusion
Buffel & Phillipson, (2019), UK [67]	**Aim**: this study explores the experiences of older residents who have lived much of their adult lives in the same locality but whose views have been largely ignored in gentrification research **Duration**: not reported **Urban setting**: 3 neighborhoods in a suburb of Manchester **Active aging element**: active aging, aging well, aging-in-place	***N*** = 106 ***Co-researchers*** ***N*** = 18 (female = 10; male = 8) **Age range** = 58–74 years ***Focus groups*** ***N*** = 58 (female = 33; male = 25) **Age range** = 60–95 years **Ethnicity** = White British (67%); White Irish (23%); White Other (7%); Black/African/Caribbean/Black British (3%) ***Interviewees*** ***N*** = 30 (female = 14; male = 16) **Age range** = 61–98 years **Ethnicity** = White British (40%); White Irish (23%); White Other (7%); Asian/Asian British (13%); Black/African/Caribbean/Black British (3%) **Target population** = older residents	**Design**: co-research; PAR **Methods**: focus groups and interviews conducted by older adults trained as co-researchers	**2 main themes emerged**: 1) Experiences of community change related to developments affecting neighborhoods, tension between those moving into the area and those living there, experiences of rejection or exclusion 2) Responses to gentrification: strategies of control related to natural neighborhood networks in terms of informal relationships that enhance well-being and shape everyday social world
Ronzi et al. (2020), England [68]	**Aim**: to explore the extent to which respect and social inclusion were promoted as the city sought to become more age-friendly and to actively involve older adults in the research process **Duration**: 1.5 months **Urban setting**: Liverpool, UK **Active aging element**: healthy aging, active aging	***N*** = 26 (female = 19, male = 7) **Age range** = 60–85 years **Ethnicity** = White British (77%), Asian British (8), Black African British (12%), Other White Background (3%) **Target population** = older person aged 60 + years	**Design**: CBPR **Methods**: photovoice, semi-structured interviews, focus group discussions	**1) Aspects of urban environments** influential of respect and social inclusion included accessibility, affordability and sociability of physical spaces. The physical and social environment contributed to a sense of exclusion. Communication and access to information were important aspects of social and digital inclusion **2) The physical environment** encompassed green and blue spaces, transportation and public facilities **3) The social environment** encompassed places to cultivate learning, art, culture, informal and formal relationships, negative age perceptions and neighborhood fragmentation
Black et al. (2015), USA [69]	**Aim**: to advance our understanding of older adults’ perceptions and the broader contributions of community residents in affecting dignity and independence in everyday interactions with older adults **Duration**: 4 months **Urban setting**: community life/community-dwelling **Active aging element**: aging well	***N*** = 484 ***Community-based forums and online participation*** ***N*** = 217 **Age range** = 12–96; 12–19 (10%), 20–44 (13%), 45–64 (39%), 65 and older (38%) **Ethnicity** = not reported ***Focus groups*** ***N*** = 51 (female = 80%; male = 20%) **Age range** = 65–98 years **Ethnicity** = White (83%), African American (15%), Other (2%) ***E-survey*** ***N*** = 216 (female = 70%; male = 30%) **Age range** = 65–96 **Ethnicity** = White (98%), African American (2%) **Target population** = residents of local geographic community and age 65 or older	**Design**: PAR **Methods**: community forums, focus groups and online surveys	**6 actionable themes of aging with dignity & independence**: 1) Meaningful involvement 2) Respect & inclusion 3) Communication & information 4) Health & well-being 5) Aging in place 6) Transportation and mobility
Annear et al. (2014), New Zealand [70]	**Aim**: to develop community-generated recommendations to inform urban environmental remediation following earthquakes in Christchurch, New Zealand, and share these with local decision makers during a participatory action research process **Duration**: 1 month **Urban setting**: Christchurch (city, 49,000 residents) **Active aging element**: active aging	***N*** = 355 ***Survey phase*** ***N*** = 355 (no other characteristics reported) ***Qualitative research phase*** ***N*** = 66 (*N* = 30 for focus group phase; female = 41; male = 25) **Mean age range** = 67–82 years **Ethnicity** = not reported **Target population** = aged 65 years and older and residing in 1 of the 12 study areas	**Design**: PAR **Method**: focus group discussions	**6 emergent themes of barriers to active aging**: 1) Loss of activity venues 2) Cancellation of meetings and events 3) Confinement and isolation 4) Fragmentation of social networks 5) Damage to transport networks 6) New hazards 1) Disruptions to activities of daily living **6 recommendations for post-earthquake redevelopment:** 1) Remediation of earthquake-affected suburbs 2) Transportation and mobility needs in earthquake-affected suburbs 3) Age and disability–friendly rebuilding 4) Safe and resilient communities 5)Resilience of support agencies 6) Access to venues for social and cultural activities
Mahmood et al. (2012), USA/Canada [71]	**Aim**: to conduct a participatory research process with community-dwelling older adults using a photovoice method to identify neighborhood physical environmental features and social aspects that influence physical activity in older adults **Duration**: 2-week photovoice activity and up to 4 training or discussion sessions **Urban setting**: 4 neighborhoods in metropolitan Vancouver and 4 neighborhoods in Greater Portland **Active aging element**: aging (discusses active aging)	***N*** = 66 **Portland** ***N*** = 32 (female = 20; male = 12) **Age range** = 65–92 **Ethnicity** = not reported **Oregon** ***N*** = 34 (female = 25; male = 9) **Age** = 65–87 **Ethnicity** = not reported **Target population**: 65 years of age or older, living in the community	**Design**: participatory action research **Method**: photovoice and discussion group	**7 themes emerged from photographs**: 1) Being safe and feeling secure 2) Getting there 3) Comfort in movement 4) Diversity of destinations 5) Community-based programs 6) Peer support 7) Intergenerational/volunteer activities
Glover et al. (2020), UK [72]	**Aim**: provide a description of a theoretically informed co-creation study to investigate what it means to maintain health and well-being in older age and how to support this in a local context. Secondly, we offer process evaluation with reflections and recommendations on practice and effective co-working **Duration**: 3 months **Urban setting**: Northern UK city **Active aging element**: healthy aging	***N*** = 14 ***Lay persons*** ***N*** = 10 (female = 7; male = 3) **Age range** = 70–79 **Ethnicity** = White British (100%) ***University members*** ***N*** = 4 (female = 4) **Age range** = 27–57 **Ethnicity** = White British (100%) **Target population** = not reported	**Design**: An iterative co-creation approach using a qualitative approach **Methods**: Project group meetings with an open structure	**The outcomes demonstrated the following key points**: 1) Develop a shared understanding of the meaning of healthy aging 2) Identify barriers and facilitators to adopting behaviors that would support the identified essential components of healthy aging 3) Make recommendations for adapting local services or developing new ones that are feasible, acceptable and sustainable
Fang et al. (2016), Canada [73]	**Aim**: how applications of community-based participatory research methods, in particular, participatory community mapping workshops (PCMWs), can be used to access experiences of place, identify facilitators and barriers to accessing the built environment and co-create place- based solutions amongst older people and service providers in a new affordable housing development in Western Canada **Duration**: not reported **Urban setting**: Senior Community Centre **Active aging element**: Positive Aging (not only aging-in-place but positive aging in the ‘right’ place)	***N*** = 54 ***Residents of affordable senior housing development*** ***N*** = 38 **Age** = over 60 **Ethnicity** = not reported ***Local service providers*** ***N*** = 16 **Age** = not reported **Ethnicity** = not reported **Target population** = residents of a seniors housing development and local service providers	**Design**: CBPR **Methods**: participatory community mapping workshops, group walks & mapping exercises	**3 key themes for establishing opportunities for positive aging-in-place**: 1) Identifying services and voicing needs 2) Opportunities for social participation 3) Overcoming cross-cultural challenges
Buffel (2018), UK [58]	**Aim**: to provide insights into the process of co-producing a research project with older residents living in low-income neighborhoods in Manchester, UK **Duration**: not reported **Urban setting**: low-income neighborhoods in Manchester, UK **Active aging element**: active aging (through the WHO age-friendly city focus)	***N*** = 86 ***Co-researchers*** ***N*** = 18 (female = 10; male = 8) **Age range** = 58–74 **Ethnicity** = White British (9); Black British (1); White Irish (4); Asian British (2); Black Caribbean (1); Black African (1) ***Interviewees*** ***N*** = 68 (female = 37; male = 31) **Average age** = 75 **Ethnicity** = minority ethnic background (32%) **Target population** = personal experiences of aging; links with different groups of older residents; good communication and listening skills	**Design**: co-production and co-research building upon partnership strategy **Methods**: training older adults as co-researchers, who then completed interviews	**Motivations to become a co-researcher**: 1) The desirability of maintaining an active post-retirement lifestyle which included a ‘busy ethic’ 2) Commitment to neighborhood change which included ‘contributing to neighborhood change’ 3) Opportunities for personal development which included ‘learning from each other’ 4) The relationship between co-researchers and interviewees which included strengths, such as peer-to-peer approach in which co-researchers could empathize with interviewees’ experiences, and weaknesses, such as co-researchers’ and interviewees’ attitudes towards aging which may be insensitive and create pressure
Brookfield et al. (2020), UK [62]	**Aim**: to further aid older adult’s involvement in environment design decisions; the aim is to provide a critical perspective on 8 ‘less traditional’ engagement techniques **Duration**: 3 years **Urban setting**: central Manchester, Hackney Wick (East London), Kirkwall (Orkney Islands) **Active aging element**: healthy aging	***N*** = 93 ***Co-design workshop*** ***N*** = 36 (no other characteristics reported) ***Design review*** ***N*** = 35 (no other characteristics reported) ***1 to 2 interactions*** ***N*** = 22 (no other characteristics reported) **Target population** = community-dwelling older adults (stroke survivors and people with dementia included in the 1-to-1 interactions)	**Design**: participatory design events (including co-design workshops) **Methods**: co-design workshops, design review events, photovoice, walking interviews, photo-elicitation, talking mats, model-making, participatory mapping, drawing. Within these methods, structured interviews & focus groups were implemented	**Valuable engagement techniques for aiding older adult involvement in environment design**: 1) Walking interviews due to immersion in the environment enabling important topics related to design to be discussed 2) Photovoice is easy to engage with and produced extensive material for design decisions 3) Interviews using photo-elicitation and ‘Talking Mats’ prompt expansive responses and are popular and novel techniques for older adults 4) Design fairs encouraged involvement due to a ‘drop-in’ approach
Aw et al. (2017), Hong Kong [74]	**Aim**: to identify, contrast and explain the continuum in which older people in the multi-ethnic diverse Asian context of Singapore participate in community and social life **Duration**: 4 months **Urban setting**: outdoor neighborhood settings **Active aging element**: active aging	***N*** = 109 ***Community focus groups*** ***N*** = 83 (female = 59, male = 22) **Median age** = 65–79 **Ethnicity** = Mixed (30%), Chinese (36%), Indian (24%), Malay (10%) ***Photovoice*** ***N*** = 19 (female = 12, male = 7) **Median age** = 60–74 **Ethnicity** = Chinese (74%), Malay (26%) ***Walkthrough spaces*** ***N*** = 7 (no other characteristics reported) **Target population** = older than 55 years old	**Design**: ethnographic approach and CBPR **Methods**: photovoice, ‘go-along’ interviews and focus group discussions	**3 ways in which older people participate in community and social life in relation to active aging**: 1) Seeking out consistent social interactions 2) Seeking expansion of social network 3) Seeking to give back to society
Salma and Salami (2020), Canada [75]	**Aim**: to elicit perceptions of aging and related needs of immigrant Muslim communities in an urban centre in Alberta **Duration**: 7 months **Urban setting**: urban centre in Alberta, Canada **Active aging element**: healthy aging, aging well	***Older adults*** ***N*** = 51 (female = 74%, male = 26%) **Age range** = 55–85 **Ethnicity** = South/East Asian (49%), Arab (26%), African (21%), Other (4%) ***Stakeholders*** ***N*** = 6 (no other demographics reported) **Target population** = being a community-dwelling individual, 55 years of age or older, self-identified as Muslim and an immigrant to Canada	**Design**: CBPR **Methods**: focus groups and individual interviews	**3 major themes capture experiences of growing old in Canada**: 1) Aging whilst living across planes 2) Negotiating access to age-supportive resources in a time of scarcity 3) Re-envisioning Islamic approaches to eldercare
Novek and Menec (2014), Canada [76]	**Aim**: to use a participatory methodology to explore older adults’ perceptions of age-friendliness and to identify priorities and barriers to making communities more age-friendly **Duration**: not reported **Urban setting**: Manitoba (city, population of 660,000) **Active aging element**: active aging, aging within community context	***N*** = 30 ***Urban*** ***N*** = 8 (female = 7; male = 1) **Age range** = 54–79 **Ethnicity** = not reported ***Rural*** ***N*** = 22 (female = 16; male = 6) **Age range** = 54–81 **Ethnicity** = not reported **Target population** = not reported	**Design**: participatory approach **Methods**: photovoice, interviews & group discussions	**1) Age-friendly features** included the physical environment, businesses and services, housing, social environment, activities and volunteering, community support and health services, and transportation **2) Contextual factors** included community history and identity, aging in rural and remote communities and environmental conditions **3) Cross-cutting themes** included independence, affordability and accessibility
Adorno et al. (2018), USA [77]	**Aim**: to examine older adults’ experiences and perspectives regarding transportation mobility **Duration**: 4 months **Urban setting**: Arlington, Texas (third largest city, major urban centre) **Active aging element**: aging well	***N*** = 60 ***Homebound*** ***N*** = 15 (53% female; 47% male) **Mean age (SD)** = 71.2 (9.45) **Ethnicity** = White (73%), African America (13%), Hispanic (13%) ***Focus groups*** ***N*** = 45 (sex not reported) **Mean age range** = 62.89–81 **Ethnicity** = White (38%), Vietnamese (24%), Hispanic (21%), African American (17%) **Target population** = Arlington residents aged 55 or older	**Design**: CBPR **Method**: semi-structured interview guides and demographics questionnaire	**4 transportation mobility barrier themes to aging well**: 1) An inadequate system: I can take you, but I can’t get you home 2) People and places: transitioning in different directions 3) Being ‘Stuck’: the political economy of transportation 4) If we’re shut out, we’re stuck in (perception of larger community being unsupportive to persons who are economically and transportation disadvantaged)
Chui et al. (2019), Hong Kong [59]	**Aim**: to explore how the process of participating in a photovoice project facilitated participants’ civic participation and to propose an empowerment-based participatory photovoice training model **Duration**: not reported **Urban setting**: local communities of participating older adults **Active aging element**: active aging	***N*** = 12 (female = 6; male = 6) **Mean age** = 66.8 (5.5) **Ethnicity** = not reported **Target population** = older adults who previously took part in photovoice training	**Design**: empowerment-based participatory training model **Methods**: focus groups to evaluate empower-based participatory photovoice training model (previously used)	**6 key themes for the photovoice process**: 1) Photovoice is an effective means of conveying older adults’ views 2) Photovoice broadens older adults’ perspectives and promotes inclusion 3) Photovoice enables knowledge acquisition and dissemination 4) Photovoice enhances older adults’ civic awareness and participation 5) Photovoice fosters a sense of confidence and empowerment amongst old adults 6) Photovoice fosters intergenerational relationships
Garvin et al. (2012), Canada [78]	**Aim**: (1) to delineate those characteristics of the built environment that are meaningfully different between summer and winter from the perspective of older adults; (2) to ascertain which of the elements of the WHO Checklist of Essential Features of Age-Friendly Cities are relevant to Edmonton seniors, and how; (3) to identify and critically evaluate additional characteristics seniors identify as particularly helpful to consider in low-density northern winter cities **Duration**: 2-week data collection and 1 focus group **Urban setting**: semi-urban & suburban neighborhoods in a northerly major metropolitan area (population over 1 million) **Active aging element**: healthy aging	***N*** = 11 ***Summer*** ***N*** = 6 (female = 4, male = 2) **Age range** = 65–84 **Ethnicity** = not reported ***Winter*** ***N*** = 5 (female = 2, male = 2) **Age range** = 55–84 **Ethnicity** = no reported **Target population** = not reported	**Design**: photo elicitation **Methods**: photo-elicited focus group method (focusing on older adults telling their story)	**Summer concerns for healthy aging included**: 1) Ramps, stairs, railings and curb cuts 2) Fear of others 3) Obstacles and broken pathways 4) Seating 5) Public transit 6) Aesthetics and cleanliness **Winter concerns for healthy aging included**: 1) Ice, snow, windrows, drainage 2) Cleanliness, litter 3) Bus shelters 4) Meeting places
Parekh et al. (2018), USA [79]	**Aim**: to explore the role of social capital (e.g. social support through indirect ties) and social cohesion (e.g. interdependent support amongst neighbors) to unravel pathways for building age-friendly communities **Duration**: 5 months **Urban setting**: urban city in Southern US **Active aging element**: aging well active aging, successful aging	***N*** = 60 (homebound = 15; classed as having a disability = 3) **Mean age** = 68 **Ethnicity** = African American (11%); Vietnamese (17%); Hispanic (15%); Caucasian (27%) **Target population** = at least 55 years of age and a current resident of the city	**Design**: CBPR **Methods**: in-depth interviews and focus groups	**3 overarching themes that link social capital, social cohesion and civic engagement**: 1) Opportunities for social cohesion & civic engagement 2) Social inclusion barriers 3) Ageism
Hand et al. (2018), Canada [80]	**Aim**: (1) to describe a combined qualitative-geospatial approach for studying of older adults in neighborhoods; (2) to investigate the qualitative-geospatial approach developed, including its utility and feasibility in exploring person–place transactions in neighborhoods **Duration**: not reported **Urban setting**: mid-sized Canadian city **Active aging element**: aging in context, aging in neighborhoods	***N*** = 14 (female = 11, male = 3) **Age range** = 66–94 **Ethnicity** = Caucasian (100%) **Target population** = aged 65 years or older and living in 1 of the target neighborhoods for at least 1 year	**Design**: CBPR and qualitative-geospatial approach **Methods**: participatory geospatial methods including narrative interviews, go-along interviews, GPS tracking with activity diary, map-based interviews	**Findings related to the unique understanding that each method contributes including**: 1) The personal experiences provided though narrative interviews, i.e. friendliness of neighborhood encourages connections to new homes, and walkability and amenities support healthy identity 2) Observing participants in the go-along interviews, i.e. how participants interact with their social environment through greeting people and dogs, chatting with strangers in shops 3) Sense of participant’s daily lives in their communities through activity/travel diaries 4) Construct further understanding of data through GPS maps and data
Verma & Huttunen (2015), Helsinki [81]	**Aim**: (1) to include the elderly as active participants in society; (2) to gain understanding of the user experience of the living environment integral to further local development **Duration**: not reported **Urban setting**: Lauttasaari (20,000 inhabitants, built environment, neighborhood under development) **Active aging element**: active aging, successful aging, aging in place	***N*** = 80 **Age range** = 64–92 **Ethnicity** = not reported ***Questionnaire*** ***N*** = 64 (female = 81%; male = 19%) ***Workshops*** ***N*** = 18 (female = 72%; male = 28%) ***Walk-throughs*** ***N*** = 18 (female = 91%; male = 9%) **Target population** = elderly residents in Lauttasaari	**Design**: participatory user study methods **Methods**: workshops, walk-throughs, Internet-based online questionnaire	**User experience of the living environment included**: 1) Social inclusion: Living alone in an unsuitable apartment can cause social isolation, and older adults were interested in housing models that enhance mutual aid and peer support 2) Services: To cope independently at home, it is important to be able to access services such as to go shopping and use public transportation 3) Green areas: Nature and the sea are important sources of well-being, but older adults expressed the desire to have more common activities and sheltered sitting places in their own yards 4) Public transportation: Lauttasaari is well connected to the city centre, and older adults were heavy users of public transportation

*CBPR* community-based participatory research, *PAR* participatory action research.

### Quality of Citizen Science and Other Participatory Approaches

The development and use of the CSAT (Table 3) highlighted the capacity for CS to further the age-friendly agenda by effectively engaging older adults to identify local urban barriers and facilitators. The CSAT also demonstrated elements of CS approaches employed in the studies that could be further strengthened to advance the level of active engagement and long-term sustainability of a study and its outcomes.

**Table 3:** Citizen Science Appraisal Tool (CSAT) to evaluate the quality of citizen science studies using a 16-question scoring system with a maximum total of 32 points (Y (Yes) = 2 points, N (No) = 0 points and ? (Unclear) = 1 point). See Appendix 3 for guidance on each question presented

Section	Question	Y	N	?
Science & research	1) Is there a clear statement of the aims, objectives or goals of the study?			
	2) Is it clear that the study used a citizen science approach?			
Leadership & participation	3) Is the degree of active engagement or participation of citizens identified clearly by the study?			
	4) Are the roles, responsibilities and type of partnership between citizens, scientists and stakeholders identified and transparent?			
Delivery & data	5) Is the extent to which citizen scientists are actively engaged or collaborate in the data collection, analysis and use/dissemination clear?			
	6) Are citizen science data limitations or biases considered by the study?			
Outcome, evaluation & open data	7) Are the main findings of the study clearly described?			
	8) Are the study’s outcomes a direct result from the data-driven strategies and solutions generated by the citizen scientists?			
	9) Do the outcomes of the study have ‘real-world’ decision making implications or impact?			
	10) Does the study report intention to track and/or tracking of long-term impacts, changes or ‘ripple effects’ of the study?			
	11) Does the study report any evaluation of citizen knowledge, attitudes, actual and/or intended behaviors?			
	12) Does the publication report any accessible dissemination plans or intentional mechanism for sharing the study and its outcomes with citizens?			
	13) Are citizens invited to review or participate in the study’s publication process?			
	14) Are the study’s results and outcomes published in an open access format and/or shared in a publicly accessible format?			
	15) Are citizen scientists acknowledged in the study’s results and publications?			
	16) Does the publication provide any critical evaluation of the study, methods and/or examination of its limitations?			

Scores will be categorised using the following scale system, adapted from Wijewardhana et al. [83] Checklist

#### Overview of Critical Appraisal Outcomes

The evaluative outcomes were categorised according to each study’s score from the 4 sections of the CSAT. Fourteen studies were categorised as medium–high (presented below in detail), 8 studies were categorised as medium and 1 study was categorised as low-medium for quality (Table 4) (see Supplementary Material 2 for CSAT outcomes and medium- and low-medium-quality studies).

**Table 4:**
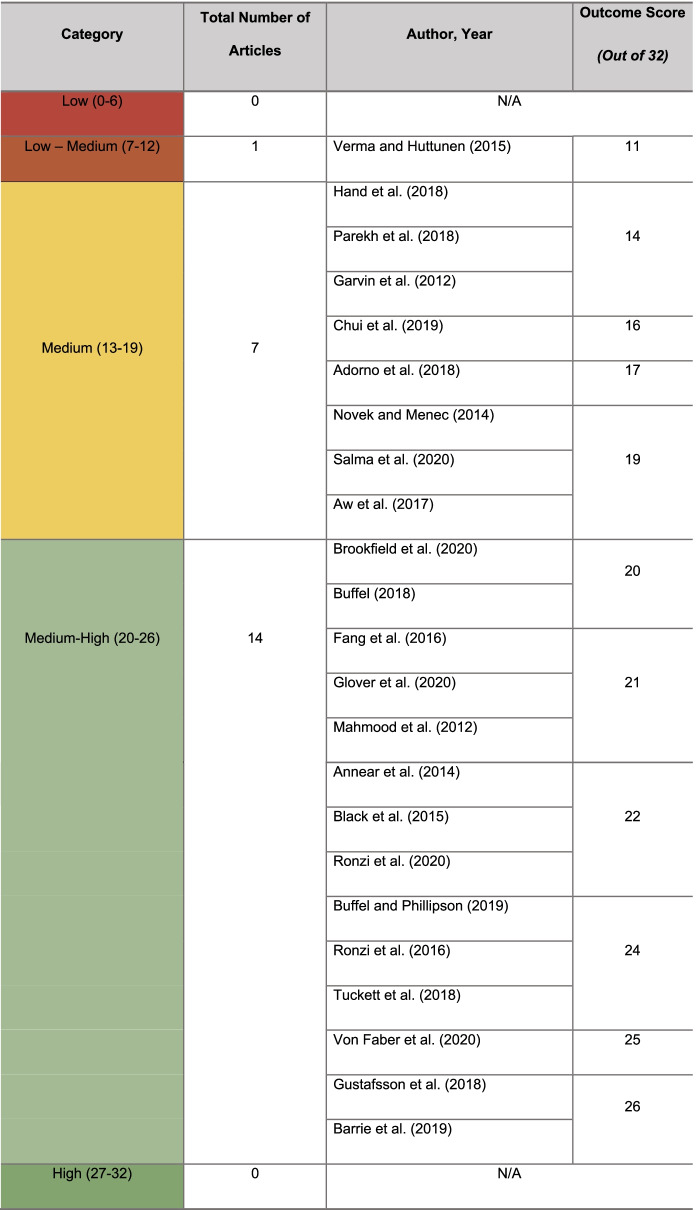
Overview of the 23 included articles and their assigned category using CSAT. A detailed explanation of scoring outcomes is available in the Supplementary Material 2

#### Medium–High Quality Studies

**Fig. 2:**
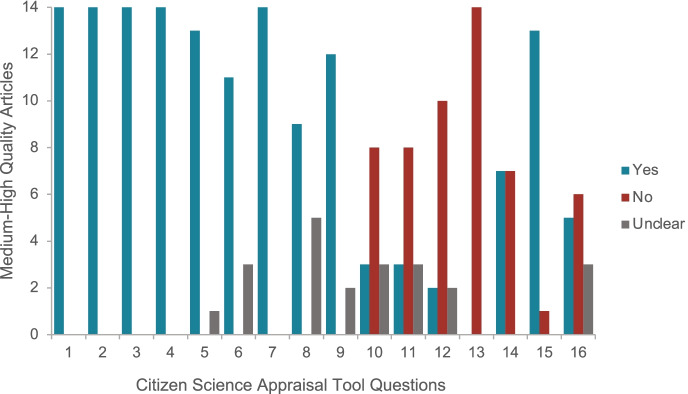
Citizen Science Appraisal Tool outcomes for medium–high-quality articles

Studies in this category (*n* = 14) scored highly in the first 3 sections of the tool (Fig. 2), suggesting key aspects to inform best practice. These studies provided a clear overview of their CS or participatory approach, the degree of active engagement of residents in all or parts of the study, clarity about the roles or types of partnership between CS and researchers, and the active engagement of CS in data collection and some analysis and/or interpretation. Thirteen of the 14 studies also acknowledged residents in their publication [58, 62–68, 70–73, 82], 12 reported outcomes that showed ‘real world’ implications or pathways (e.g. pathways towards cultural and policy implications) [62–65, 67–73, 82], and 9 demonstrated outcomes that were generated directly by residents [58, 64, 65, 67–71, 73].

Amongst studies rated as ‘medium–high’, the levels of resident engagement included partnering in all stages of the research (*n* = 7), guiding the research design (*n* = 7), actively collecting data (*n* = 14) and analyzing (*n* = 7) or member checking data (*n* = 10). Two studies demonstrated a ‘by the people’ engagement [64, 65], 1 demonstrated ‘by the people’ and ‘for the people’ [70] and 11 demonstrated a combination of ‘by the people’ and ‘with the people’ [58, 62, 63, 66–69, 71–73, 82]. Studies presented strong elements of engagement by actively involving residents to partner in all stages of the research process [65] or to co-design [62], direct [73], co-create [64] or co-conduct [58, 67] the research methods. Some studies also had member checking of data [58, 63, 65–67, 70–73, 82] or guidance from resident advisory groups [64, 69, 70].

### Community-Identified Urban Environment Characteristics Influencing Active Aging

The urban environment characteristics were identified across Canada (*n* = 6) and/or the USA (*n* = 4), the UK (*n* = 5), Australia (*n* = 2), Hong Kong (*n* = 1), Netherlands (*n* = 1), Sweden (*n* = 1) and Finland (*n* = 1) and encompassed a range of cities (*n* = 9), urban or suburban neighborhoods and residential areas (*n* = 8), urban districts or centres (*n* = 2), towns (*n* = 1) and public green spaces (*n* = 1).

#### Urban Environment Barriers to Active Aging

**Fig. 3:**
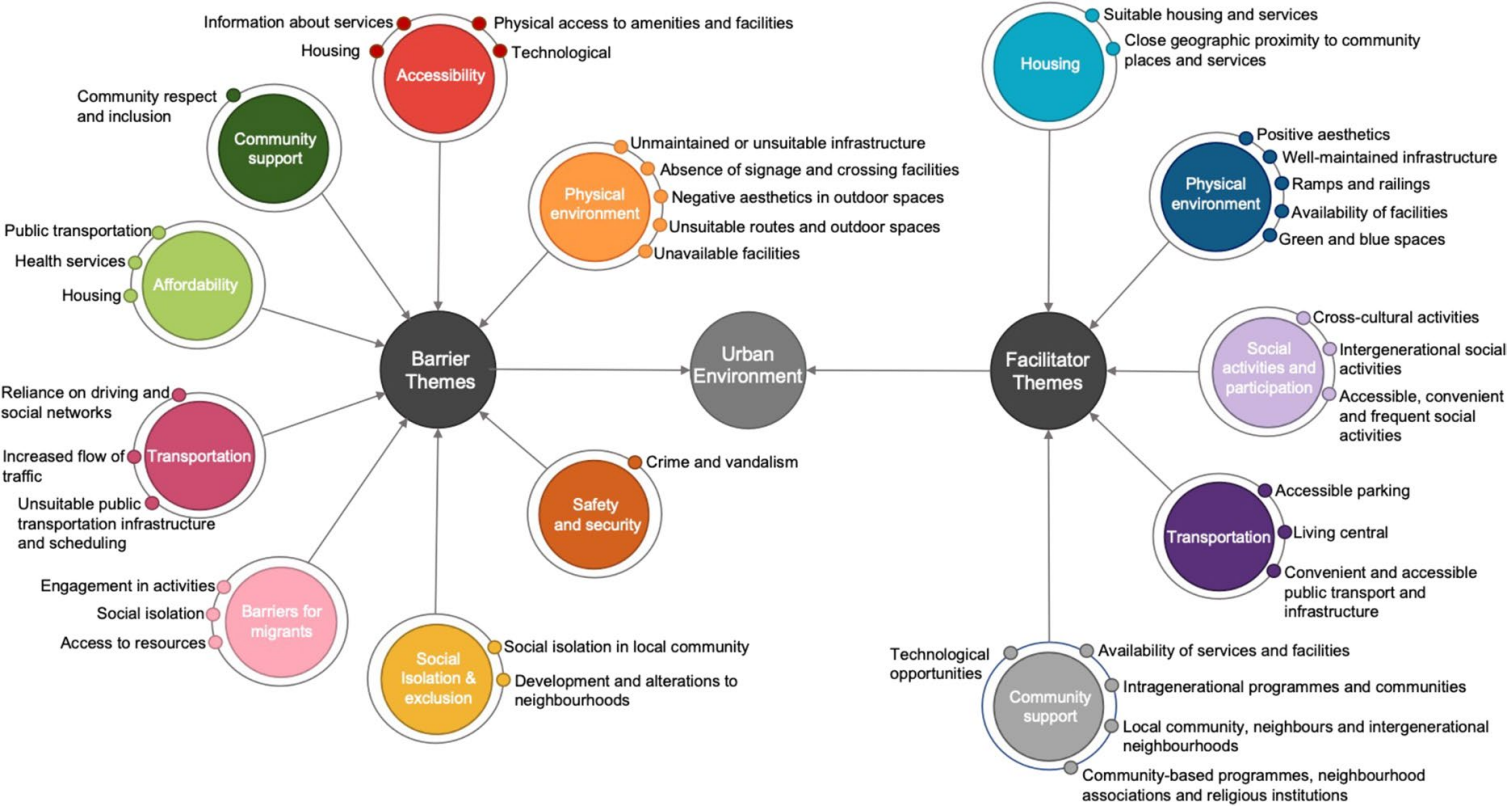
Urban environment barriers and facilitators. Smaller circles indicate subthemes

Eight themes relating to urban environment barriers to active and healthy aging (Fig. 3) were identified (see Appendix 1). These included accessibility (*n* = 14), physical environment (*n* = 13), transportation (*n* = 10), affordability (*n* = 8), social isolation and exclusion (*n* = 6), community support (*n* = 5), barriers for migrants and cross-cultural communities (*n* = 5) and safety and security (*n* = 4).

Accessibility was the most commonly identified barrier (*n* = 14), highlighting the lack of physical and disability–friendly access to local buildings, services and facilities as a key impediment to active aging. Accessible information about services, accessible housing and the advancement of modern technology making devices difficult to use were also included in this theme. Physical environment was another common barrier (*n* = 13), where residents documented a lack of well-maintained outdoor environments, the presence of physical obstacles and the absence of street crossing elements as barriers. Such barriers were particularly prominent during winter and for people with visual and physical impairments. Lack of available toilets and other facilities, negative aesthetics in outdoor spaces and unsuitable routes also negatively impacted active aging according to residents.

#### Urban Environment Facilitators to Active Aging

Five themes relating to urban environment facilitators (Fig. 3) were identified by residents to be positively associated with active and healthy aging (see Appendix 1). These included community support (*n* = 14), physical environment (*n* = 11), social activities and participation (*n* = 10), transportation (*n* = 7) and housing (*n* = 4).

Community support was the most community-citied facilitator (*n* = 14), stressing the importance of local communities and neighborhoods, particularly intergenerational ones, providing support and encouraging social activities. The availability of shops, cafes and public places such as libraries was a facilitator and useful for developing social links during winter. The presence of network associations, senior-specific programmes and religious institutions were all identified as sources of support that promote health, well-being and independence. Physical environment was also a commonly cited facilitator (*n* = 11), underscoring the importance of seating, positive aesthetics such as cleanliness and well-maintained infrastructure in both summer and winter to facilitate active aging.

### Mapping of Community-Identified Urban Barriers and Facilitators against the WHO Checklist of Essential Features of Age-Friendly Cities

#### Aligning and Interconnecting Themes and Features

**Fig. 4:**
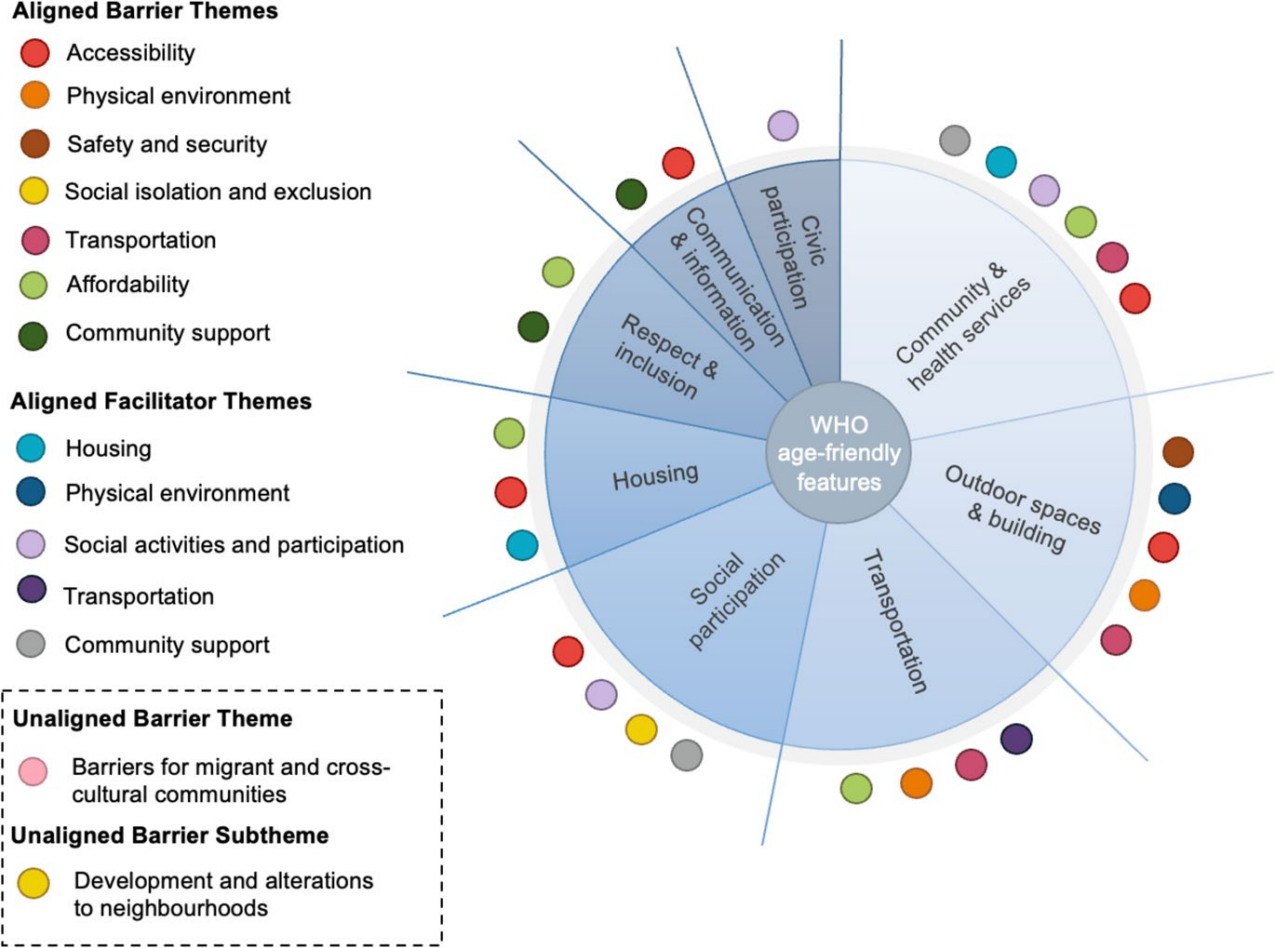
Visual representation of the barriers and facilitators mapped against the features presented by the WHO Checklist of Essential Features of Age-Friendly Cities [37]

The 13 themes ( Fig. 3) significantly aligned with the WHO’s 8 features and descriptors presented on the essential features of age-friendly cities checklist [37] (Fig. 4) (see Appendix 2). Physical environment barriers and facilitators, transportation barriers, safety and security, and accessibility were themes from this review that aligned with 9 out of the 10 WHO descriptors of the outdoor spaces and building features. Similarly, the themes of accessibility, social isolation and exclusion, social activities and participation, and community support aligned with 8 out of 11 WHO descriptors of the social participation feature. Both barrier and facilitator themes also aligned with 9 out of 12 WHO descriptors of the communication and information feature and with 10 out of 15 WHO descriptors of the transportation feature. The alignment of themes to the WHO features and descriptors indicates the ongoing relevance of bringing together local community–identified features with those presented on authoritative checklists to determine key features for developing age-friendly environments.

#### Unaligned Themes and Features

Despite the close alignment of WHO features and community-generated urban characteristics, the CS process did elucidate additional themes regarding age-friendliness of urban environments. Most significant amongst these were *barriers for migrant and cross-cultural communities*. This theme highlighted how ethnicity and cultural differences can negatively impact engagement in activities outside of the home due to differences in cultural norms and social exclusion. Members of culturally, ethnically and linguistically diverse minorities themselves highlighted poor access to transportation, lack of social platforms for civic engagement, lack of suitable care facilities and lack of support from the government.

Similarly, *developments and alterations to neighborhoods*, which was a subtheme of social isolation and exclusion, did not align with the features or descriptors of the checklist. Exploration of this subtheme indicated that physical alterations to neighborhoods, the presence of new builds and frequent relocation of neighbors negatively impacted social connections, community cohesion and the feeling of ‘locality’ for older adults. Considering the needs of migrant and cross-cultural communities and the impacts that new built environment developments can have on the risk of social isolation and exclusion are new dimensions that need to be further encompassed into the age-friendly agenda in order to strengthen this approach.

## Discussion

Having systematically reviewed the literature on urban environments and active aging, we highlight three areas for consideration: (1) the importance of a socio-ecological perspective on the local urban characteristics associated with active and healthy aging, (2) expanding the WHO age-friendly cities agenda in light of local urban characteristics and (3) strengthening the field of CS to further its potential in capacity building the age-friendly agenda.

### Socio-ecological Perspective of Urban Environment Characteristics

Accessibility, physical environment features and the presence of community support were key urban characteristics associated with active and healthy aging and require further consideration when developing age-friendly environments. Using a socio-ecological approach, which incorporates personal, environmental, social, cultural, economic and political domains [4, 85–89], can provide a comprehensive indication of the features that influence health and active living [85, 90]. This approach also has been built into a systems perspective [3, 91], which strengthens the understanding that ecosystems are multi-levelled and interacting systems governed by change and adaptations [92, 93]. This review employed a socio-ecological perspective to conceptualise the urban environment characteristics. These are presented by the themes as part of an interconnected system in which there are multiple domains and complex interrelationships between individuals and their environments [94]. Rather than considering these urban characteristics in relative isolation, we provide two examples of interconnected systems relating to affordable housing and social activities.

#### Affordable and Accessible Housing

The review highlighted interconnected barriers and facilitators for suitable, accessible and affordable housing close to community services, which have previously been linked through a socio-ecological perspective [92, 95–97]. The interpersonal relationships with neighbors, physical location or material quality of a house, and social interactions between who is being housed and the suitability of the house, as well as how these interactions change over time, all interconnect and influence the individual [92, 95, 98]. The economic and material resources and the development and implementation of suitable housing policy are also identified by the literature as connected and impact the presence of suitable and affordable housing [92, 97, 98]. To promote affordable and accessible housing in an age-friendly context, the outcomes of this review indicate a need to consider the interconnection between an individual’s needs and required services, the affordability and accessibility of housing and the geographic proximity to the wanted services and facilities. This can shift the development of housing away from considering these domains and barriers in isolation [98] and instead addressing the multiple interconnected housing influences present in order to develop suitable age-friendly housing.

#### Social Activities and Participation, and Social Isolation

Social activities and participation, and social isolation were themes that interconnected amongst the personal, environmental and social domains of urban environments. Across a number of studies, engagement in social activities is influenced by personal factors such as mobility [99], motivation and social values [100, 101], as well as various aspects of the environmental and policy domains, which influence the presence of suitable environments [102], resources and places to participate socially [101, 103]. For older adults, engaging in activities is also shown to be influenced by social factors including the presence of a social network, neighbors and/or a sense of community [104, 105]. Individual characteristics and influential factors across social and environmental domains need to be considered in order to provide spaces that facilitate age-friendly social participation and accessible activities. In particular, the CS processes enabled residents to identify intergenerational and cross-cultural activities and spaces where neighbors and local communities can build community cohesion and support social participation as key facilitators.

### Strengthening the Age-Friendly Cities Initiative

Whilst the WHO Checklist of Essential Features of Age-Friendly Cities provides a useful and universal guide to assessing and implementing features, such work can be supported by more attention to local urban characteristics. CS has made clear that the subtheme ‘*neighborhood developments and alterations*’ and the theme ‘*barriers faced by migrant and cross-cultural communities*’ require further exploration. Recognising contextual importance by considering the impact of neighborhood changes on social isolation and identifying the multiple barriers faced by ethnically, culturally or linguistically diverse minorities are important for strengthening the age-friendly agenda. Whilst these two themes are considered below, it is important to recognise that strengthening this agenda requires extension to populations experiencing social exclusion such as minority ethnic groups, refugees and individuals from LGBTQ communities to inform the design of future initiatives aiming to develop inclusive age-friendly environments [106, 107].

#### Neighborhood Developments and Alterations

Physical alterations to neighborhoods, new builds and rotation of neighbors were identified as negatively impacting social connections and community cohesion. Changing and gentrifying neighborhoods can cause experiences of social isolation, insecurity and vulnerability, challenge a sense of belonging, and negatively impact mental health [67, 108, 109]. Although neighborhood changes are experienced differently based on the contexts and types of residences in which individuals live, they have been found to negatively impact social and community cohesion, making it important to address the impacts these changes may have on older adults [66, 110, 111]. As the immediate neighborhood environment is also vital for older adults and their social support, particularly those who are vulnerable or have limited mobility [112], the developments and alterations to neighborhoods need to be further assessed in the planning and implementation of age-friendly initiatives.

It is important to consider the impacts of changing neighborhoods on older adults, especially for those who wish to remain at home or in their local neighborhoods [109], and evaluate the effect age-friendly features which in some cases have not had a real and meaningful impact [113]. Modifying the WHO guidelines to address alterations to neighborhoods that may impact older adults could enhance the social structures and community cohesion present within urban communities [108] and could lessen the physical, social and cultural consequences resulting from these changes [111].

#### Barriers Faced by Migrant and Cross-Cultural Communities

Ethnicity, cultural differences and language barriers faced by migrant and cross-cultural communities were identified to be associated with social isolation, access to resources and support from governing bodies. Aging in cities can be challenging for older adults who migrate [114]; inequalities place these individuals at an increased risk of social exclusion [115]. Age-friendly city initiatives therefore need to be tailored to local situations and serve diverse communities in order to address the context-specific factors that contribute to social isolation. For example, migration pressure, language issues, access to and lack of knowledge about existing resources and community programmes are all barriers that have been previously identified [116, 117] and should be taken into consideration.

It is currently unclear if age-friendly programmes effectively address the needs of older individuals from diverse ethnic and cultural backgrounds [118]. Better understanding of urban experiences of older adults from diverse ethnic communities is needed in order to identify the inequalities and influence of racism faced by individual ethnic groups [106, 119]. To address these challenges, the use of CS approaches requires further strengthening, which is discussed in the following section.

### Strengthening Citizen Science Approaches

To further the potential of CS in strengthening age-friendly initiatives, the CSAT identified that greater attention needs to be paid to (1) the diversity of approaches within such research methodologies; (2) the degree to which they are contributory (*for the people*), collaborative (*with the people*) and/or co-produced (*by the people*); (3) their potential unintended consequences; and (4) the sustainability of outcomes.

An absence of resident engagement in the scientific processes, particularly as they relate to informing study design or hypotheses, is a current gap in CS projects [19, 120]. This finding was reflected in the reviewed studies, stressing the need for further engagement of older adults in the planning and research design [68, 69, 71–73, 82] and data analysis [63, 66, 68, 69, 71, 72]. A recent analysis of interviews exploring participant engagement in CS projects reported an absence of engagement in informing project design or hypotheses [120]. However, increasing opportunities for engagement in multiple processes is an ideal [22] achieved through co-creation [121, 122] or a ‘by the people’ approach [21]. This more comprehensive form of engagement provides the greatest potential for developing and implementing relevant environment and social changes [19, 123], attributed to residents embedding their knowledge into design and dissemination, and enabling a project to develop in the cultural context of a local community [123, 124].

Studies have found that predominantly well-educated people from White ethnic groups rather than from minority ethnic groups engage with CS [44, 125, 126]. This has been linked to a lack of sense of belonging in areas where White ethnic backgrounds are a majority [127], differing volunteering motivations [128] and financial or educational barriers to participation [44, 126]. The studies included in this review engaged residents across a range of populations, enabling the identification of diverse urban barriers pertaining to aging. Without representative engagement in CS, the beneficial impacts and translatability of outcomes can be reduced [129], requiring consideration of effective strategies to foster opportunities for engaging migrant and diverse ethnic communities. This includes recognising barriers to engagement and presenting new pathways to engaging the views, beliefs and practices of underrepresented groups [125, 126]. For example, ensuring that CS and public engagement tools are in multiple languages can reduce such barriers to inclusion [44, 130].

For future studies to strengthen their levels of co-production, the following characteristics by Hidalgo et al. [19] should be considered: (1) co-defining and focusing on real-world issues, (2) using shared language and visual materials, (3) building an equal and collaborative research community, (4) including participatory meetings led by suitable individuals who can facilitate co-creation, and (5) providing participatory tools and accessible means of communication for disseminating outcomes.

Capturing effective dissemination of results and long-term sustainability are also current challenges amongst CS projects [84]. This was identified in the reviewed studies, with a lack of accessible dissemination plans and tracking of long-term changes or ripple effects emanating from the CS activities. Tracking long-term outcomes is a key for identifying sustainability and momentum of a project once it has finished, particularly in the physical and social environments [17, 33, 84]. Long-term observation can showcase a commitment to maintain longitudinal outcomes [131], such as ‘ripple effects’ where citizen scientists continue to advocate for neighborhood improvements after a project has ended [132]. One systematic method for capturing the multi-level impacts of CS is called ripple effects mapping [133]. This qualitative method, used originally in the US Cooperative Extension field, involves citizen scientists and other stakeholders visually mapping the cumulative trajectory of intervention-related activities and outcomes—both expected and unexpected—over time [134].

To the best of our knowledge, the CSAT is a novel appraisal tool that can effectively encompass and evaluate the quality of CS research processes, the level of engagement employed, data and long-term sustainability. The CSAT can contribute to the field of CS by guiding and strengthening the development, implementation and evaluation of future CS studies. Although the tool was designed for CS approaches in the urban and aging research field, it shows universality and can evaluate other participatory approaches across multiple disciplines. Further, the CSAT can guide researchers to foster and support citizen-driven strategies and outcomes that encompass community insights and have sustainable and real-world relevance. Whilst not within the tool’s remit, it is important for CS projects employing the tool to consider the impact of their societal and scientific outcomes if diversity or representativeness of engagement across a community is missing [44]. This includes recognising barriers to engagement faced by different ethnic groups and biases from underrepresenting or overrepresenting different groups [126]. Previous literature has identified CS approaches for greater equality in engagement [84, 135] and should be considered in detail.

### Strengths and Limitations

Due to the broad nature of CS [136], the limited capacity of the keywords to identify all relevant studies was a potential liability. The chosen keywords may not have captured all CS or other participatory studies, for example the work by Buman et al. [137] and Winter et al. [138], and especially those with creative naming or novel approaches. Secondly, although the CSAT was employed to identify good quality studies, selection bias may be present. For example, requiring the mean age of citizen scientists to be at least 60 years would have led to the omission of articles involving intergenerational groups of citizen scientists (e.g. older adults and youth [132]) that did not separate outcomes based on age or described CS work targeting older adults [17].

Despite these limitations, this review followed a systematic protocol guided by the PRISMA checklist to systematically review a large volume of literature (11,311 articles), which embodies a diverse array of articles. The chosen databases also provided a diversity of health, environmental, social science and gerontology research. Furthermore, the protocol, keywords and systematic processes were informed by a multidisciplinary team of consultants in relevant fields, who also contributed to the development and implementation of the CSAT.

## Conclusion

This systematic scoping review identified a range of urban barriers and facilitators identified by older adults engaged in CS or other participatory approaches. The interconnectedness of the barriers and facilitators was presented using a socio-ecological perspective, reflecting the need to consider the multi-level influences and associations present when developing local age-friendly environments locally. The findings also identified a need for nuanced and culturally specific approaches to increasing feelings of social inclusion and accessibility of resources for migrant communities to strengthen the age-friendly agenda. Future studies can use the CSAT to guide their CS approach and incorporate best practices into their design and methods so that processes, strategies and outcomes are co-produced with residents to shape the future of their local urban environments.

## Supplementary Information

Below is the link to the electronic supplementary material.

Supplementary file1 (PDF 326 KB)

Supplementary file2 (PDF 56 KB)

Supplementary file3 (PDF 392 KB)

## Data Availability

The data that support the findings of this study are available in the appendices and supplementary material and from the corresponding author upon reasonable request.
