# Supplementary material for: The Role of Urban Environments in Promoting Active and Healthy Aging: A Systematic Scoping Review of Citizen Science Approaches
Source: J Urban Health. 2022 May 19;99(3):427–56. doi: 10.1007/s11524-022-00622-w (PMC9187804; doi:10.1007/s11524-022-00622-w)
Supplement: Supplementary file 1 — Supplementary file1 (PDF 326 KB) [file 11524_2022_622_MOESM1_ESM.pdf]

The role of urban environments in promoting active and healthy ageing: A systematic scoping review of citizen science approaches Appendices (1, 2 and 3)

Systematic Scoping Review: Appendices

Appendix 1

Table I Urban environment barriers to active ageing

Theme	Sub-themes	First order themes	No of Articles	Reference
Accessibility	Physical access to amenities and facilities	1. Physical and disability-friendly accessibility to local buildings<br>2. Health Services<br>3. Community services<br>4. Shopping facilities<br>5. Senior centres<br>6. Activities & social gatherings	11	[1-10]

	Technological barriers	Development of modern technology negatively impacts availability and accessibility of online information and availability of physical information	5	[3, 9, 11-13]
	Information about services	Services and organisations offering activities do not communicate or signpost information	4	[4, 9, 11, 12]
	Housing	Lack of accessible housing	4	[2, 4, 9, 14]
Affordability	Health Services	Costs of health services too high and causing financial hardship and concern, especially for those ageing-in-place	6	[1, 4, 11, 12, 15]
	Housing	Lack of affordable housing creates a financial barrier, particularly for ageing-in-place	3	[2, 4, 11, 14]
	Public Transportation	High costs of public transport	4	[4, 11-13]

Community support	Community respect and inclusion	Community unsupportive, disrespectful and devalue older adult contributions	5	[1, 5, 8, 11, 13]
Barriers for migrants & cross-cultural communities	Engagement in activities	Ethnicity or differences in cultural norms prevents or negatively impacts engagement in activities outside of home	3	[2, 15, 16]
	Social Isolation	Cultural differences and language barriers increase social isolation	3	[2, 5, 15]
	Access to resources	Poor access to transport, social platforms for civic engagement, suitable care facilities, or support from government amongst ethnic, culturally and linguistically diverse minorities.	3	[1, 5, 15]

Physical environment barriers	Unmaintained or unsuitable infrastructure	Poorly maintained infrastructure, pavements and presence of obstacles, particularly during winter	8	[4, 6, 9, 10, 17-20]
	Absence of signage and crossing facilities	Lack of crossing facilities and signage for wayfinding and traffic related safety, particularly a danger for those with visual and physical impairments	8	[3, 6, 9-11, 18-20]
	Negative aesthetics in outdoor spaces	Unclean, disorderly, and displeasing physical environment	6	[9, 13, 18-20]
	Unavailable facilities	Lack of suitable toilets, facilities for drinks, benches & suitable seating, particularly in summer	8	[6, 8, 9, 12, 13, 17, 18, 20]
	Unsuitable routes & outdoor spaces	Poor walkability, unsafe and inaccessible routes or outdoor spaces	3	[6, 9, 11]
Safety & security	Crime & Vandalism	Reduces feeling of safety and time spent outdoors in urban areas	4	[4, 6, 13, 19]

Social isolation & exclusion	Social Isolation in local community	Lack of connections & activities with neighbours or local community contributes to social isolation	3	[2, 9, 12]
	Developments & alterations to neighbourhoods	Physical alterations, new builds, & rotation of neighbours negatively impact social connections, community cohesion & 'locality'	3	[13, 14, 19]
Transportation	Unsuitable public transport infrastructure, system & scheduling	Lack of public transport, physical accessibility to transportation and unsuitable infrastructure a barrier to accessing services, facilities & social activities	9	[1, 3-6, 9, 11, 13, 18]
	Reliance on driving and social networks	Lack of public transportation creates pressure for driving and reliance on social networks	2	[1, 11]
	Increased flow of traffic	Producing unsafe spaces and hazards difficult to navigate	4	[3, 6, 11, 19]

The role of urban environments in promoting active and healthy ageing: A systematic scoping review of citizen science approaches Appendices (1, 2 and 3)

Table II Urban environment facilitators to active ageing

**Theme**	**Subtheme**	**First order themes**	**No of Articles**	**Reference**
Community Support	Availability of services & facilities	Availability of shops, cafes & 'third places' such as libraries, particularly useful during winter and for social links	9	[4, 6, 9, 10, 13-15, 18, 19]
	Community-based programmes, neighbourhood associations and religious institutions	Encourages and provides support to older adults	5	[4-6, 14, 16]
	Intragenerational programmes and communities	Senior-specific programmes & community of people the same age promotes health, well-being & independence	3	[2, 4, 11]

	Local community, neighbours, and Intergenerational neighbourhoods	Provide support for older adults, particularly for ageing-in-place, and encourage social activities with others	7	[4, 6, 9-11, 14, 16]
	Technological Opportunities	Availability of computers helpful for accessing support & information	2	[11-13]
Housing	Close geographic proximity to community places & services	proximity to services, facilities and transportation services provides opportunities for community engagement, accessibility & reduces isolation	3	[2, 4, 10]
	Suitable housing and services	Age-friendly homes, assisted living and home care services are beneficial and promote well-being	3	[4, 10, 14]
	Positive Aesthetics	Aesthetics, cleanliness & positive living environments	6	[2, 4, 6, 9, 17, 18]

The role of urban environments in promoting active and healthy ageing: A systematic scoping review of citizen science approaches Appendices (1, 2 and 3)

Physical Environment Facilitators	Availability of facilities	Availability of toilets, drinking facilities and sufficient seating & benches for places to rest and socialise, especially during summer	8	[4, 6, 7, 9, 13, 17, 18, 21]
	Well-maintained infrastructure	Well-maintained paths enable walking and mobility, both in summer and winter	7	[6, 7, 9, 10, 13, 18, 21]
	Green & blue recreational spaces	Parks, public green spaces, gardens, and seashore are valued recreational spaces	6	[6, 9, 10, 13, 17, 19]
	Ramps & Railings	Enable mobility in outdoor spaces	3	[4, 6, 18]
Social activities and participation	Accessible, convenient & frequent social activities	Encourage contribution, meaningful involvement, social connections, & reduces isolation, particularly volunteering activities	9	[2, 4-6, 10-12, 16, 19]
	Intergenerational social activities	Availability of intergenerational activities encourage connection with others	3	[6, 11, 19]

	Cross-cultural activities	Availability of cross-cultural social activities beneficial for migrant communities	4	[2, 10, 15, 16]
Transportation	Convenient, affordable and accessible public transport and infrastructure	Availability of public and community transport vital for independence, transportation mobility and ageing-in-place	6	[1, 6, 8, 11, 13, 18]
	Living Central	Living central to transportation services beneficial for accessibility	2	[1, 8]
	Accessible parking	Low cost and accessible parking enables transport mobility and time spent in recreational spaces	2	[4, 6]

The role of urban environments in promoting active and healthy ageing: A systematic scoping review of citizen science approaches Appendices (1, 2 and 3)

Appendix 2

Table II Urban environment themes from the 20 included studies that map or add to the WHO Essential Features Checklist of Age-friendly Cities. *Note: (1)Green = Facilitator themes; (2) Red = Barrier themes; (3) The columns greyed out did not have any themes present in the 20 studies that matched the WHO features or description*

WHO Essential Features of Age-Friendly Cities	WHO Description of age-friendly features Description (WHO, 2007)	Theme	Subtheme	First order theme	Refs
Outdoor spaces and buildings	***Barriers***				
	Clean & pleasant public areas	*Physical environment barriers*	Negative aesthetics in outdoor spaces	Unclean, disorderly, and displeasing physical environment	[7, 9, 18, 19]

	Seating is well-maintained, safe & sufficient<br>Public toilets are sufficient in number, clean, accessible & well-maintained		Unavailable facilities	Lack of suitable toilets, facilities for drinks, benches & suitable seating, particularly in summer	[6, 8, 9, 12, 13, 17, 18, 20]
	Pavements are well-maintained, free of obstructions, reserved for pedestrians, non-slip, wide enough for wheelchairs, dropped curbs at road level.		Unmaintained or unsuitable infrastructure	Poorly maintained infrastructure, pavements and presence of obstacles, particularly during winter	[4, 6, 9, 17-20]
			Unsuitable routes & outdoor spaces	Poor walkability, unsafe and inaccessible routes or outdoor spaces	[6, 9, 11]

	Pedestrian crossings are sufficient in number, safe, non-slip, visual & audio cues, adequate crossing times		Absence of signage and crossing facilities	Lack of crossing facilities and signage for wayfinding and traffic related safety, particularly a danger for those with visual and physical impairments	[3, 6, 9, 11, 18-20]
	Drivers give way to pedestrians.	*Transportation*	Increased flow of traffic	Producing unsafe spaces and hazards difficult to navigate	[3, 6, 11]
	Outdoor safety promoted through lighting, police patrols & community education.	*Safety & Security*	Crime & Vandalism	Reduces feeling of safety and time spent outdoors in urban areas	[4, 6, 19]

	Services are accessible & situated together	*Accessibility*	Physical access to amenities and facilities	1. Physical and disability-friendly accessibility to local buildings<br>2. Health Services<br>3. Community services<br>4. Shopping facilities<br>5. Senior centres<br>6. Activities & social gatherings	[1-9]
	Special customer service arrangements for older adults				
	Facilitators				
	Clean & pleasant public areas	*Physical environment facilitators*	Positive Aesthetics	Aesthetics, cleanliness & positive living environments	[2, 4, 6, 9, 17, 18]

			Green & blue recreational spaces	Parks, public green spaces, gardens, and seashore are valued recreational spaces	[6, 9, 17]
	Seating is well-maintained, safe & sufficient		Availability of facilities	Availability of toilets, drinking facilities and sufficient seating & benches for places to rest and socialise, especially during summer	[4, 6, 9, 13, 17, 18, 20, 21]
	Pavements are well-maintained, free of obstructions, reserved for pedestrians, non-slip, wide		Well-maintained infrastructure	Well-maintained paths enable walking and mobility, both in summer and winter	[6, 7, 9, 18, 21]

	enough for wheelchairs, dropped curbs at road level.		Ramps & Railings	Enable mobility in outdoor spaces	[4, 6, 18]
	Public toilets are sufficient in number, clean, accessible & well-maintained		Availability of facilities	Availability of toilets, drinking facilities and sufficient seating & benches for places to rest and socialise, especially during summer	[4, 6, 7, 9, 13, 17, 18, 21]
	Services are accessible & situated together.		Ramps & Railings	Enable mobility in outdoor spaces	[4, 6, 18]
	Unmatched				
	Cycle paths separate from pavements & walkways				
Transportation	***Barriers***				

	Public transport costs are consistent, clear & affordable.	*Affordability*	Public Transportation	High costs of public transport	[4, 11, 12]
	Public transport is reliable & frequent.	*Transportation*	Unsuitable public transport infrastructure, system & scheduling	Lack of public transport, physical accessibility to transportation and unsuitable infrastructure a barrier to accessing services, facilities & social activities	[1, 3-6, 9, 11, 18]
	City areas & services are accessible by public transport, with good connections, well-marked routes.	*Transportation*	Unsuitable public transport infrastructure, system & scheduling	Lack of public transport, physical accessibility to transportation and unsuitable infrastructure a barrier to accessing	[1, 3-6, 9, 11, 18]

				services, facilities & social activities	
	Transport stops & stations are convenient, accessible, safe, clean, well-lit, well-marked, adequate seating & sheltered.		Unsuitable public transport infrastructure, system & scheduling	Lack of public transport, physical accessibility to transportation and unsuitable infrastructure a barrier to accessing services, facilities & social activities	[1, 3-6, 9, 11, 18]
	Voluntary transport is available when public transport is limited.		Reliance on driving and social networks	Lack of public transportation creates pressure for driving and reliance on social networks	[1, 11]

	Traffic signs & intersections are visible.	*Physical environment barriers*	Absence of signage and crossing facilities	Lack of crossing facilities and signage for wayfinding and traffic related safety, particularly a danger for those with visual and physical impairments	[3, 6, 7, 9, 11, 18, 19]
	Facilitators				
	Public transport is reliable & frequent.<br>Specialised transportation available for disabled people<br>Voluntary transport is available when public transport is limited.	*Transportation*	Convenient and accessible public transport and infrastructure	Availability of public and community transport vital for independence, transportation mobility and ageing-in-place	[1, 6, 8, 11, 13, 18]

	Parking & drop-off areas are sufficient, safe & convenient, with priority for people with special needs.		Accessible parking	Low cost and accessible parking enables transport mobility and time spent in recreational spaces	[4, 6]
	Unmatched				
	Vehicles are clean, well-maintained & not overcrowded.				
	Drivers stop at designated areas to facilitate boarding				
	Accessible information is provided about transport.				
	Taxis are accessible & affordable.				

	Roads are well-maintained, free of obstructions.				
Housing	Barriers				
	Housing is sufficient, available, in safe areas & close to services & the community	*Accessibility*	Housing	Lack of accessible housing	[2, 4, 9, 14]
	Sufficient & affordable home maintenance and support is available.	*Affordability*	Housing	Lack of affordable housing creates a financial barrier, particularly for ageing-in-place	[2, 4, 11, 14]
	Sufficient & affordable housing for frail & disabled older people is provided with appropriate services.				

	Facilitators				
	Housing is sufficient, available, in safe areas & close to services & the community	*Housing*	Close geographic proximity to community places & services	Close proximity to services, facilities and transportation services provides opportunities for community engagement, accessibility & reduces isolation	[1, 2, 4, 8]
	Sufficient & affordable housing for frail & disabled older people is provided with appropriate services.	*Housing*	Suitable housing and home services	Age-friendly homes, assisted living and home care services are beneficial and promote well-being	[4, 14]
	Unmatched				

	Housing is well-constructed & provides safe & comfortable shelter.				
	Public & commercial rental housing is clean, well-maintained & safe.				
	Barriers				
Social participation	Good information is provided about activities & events.	*Accessibility*	Information about services	Services and organisations offering activities do not communicate or signpost information	[4, 9, 11, 12]
	There is a consistent outreach to include people at risk of social isolation.	*Social isolation & exclusion*	Social Isolation in local community	Lack of connections & activities with neighbours or local community	[2, 9, 12]

				contributes to social isolation	
	**_Facilitators_**				
	Venues for activities/events are convenient, accessible, well-lit & easily reachable by public transport.	*Social activities, participation & network*	Accessible, convenient & frequent social activities	Encourage contribution, meaningful involvement, social connections, & reduces isolation, particularly volunteering activities	[2, 4-6, 11, 12, 16, 19]
	Wide variety of activities are offered that appeal to a diverse population of older people.	*Social activities, participation & network*	Intergenerational social activities	Availability of intergenerational activities encourage connection with others	[6, 11, 19]
			Cross-cultural activities	Availability of cross-cultural social activities	[2, 16]

				beneficial for migrant communities	
	Gatherings including older people are held in various local community spots.	*Community Support*	Availability of services & facilities	Availability of shops, cafes & 'third places' such as libraries, particularly useful during winter and for social links	[4, 6, 9, 14, 18]
	Unmatched				
	Activities/events can be attended alone or with companions.				
	Activities are affordable with no additional costs.				
	Barriers				

Respect and inclusion	Older people are recognised by the community for their contributions.	*Community Support*	Community respect and inclusion	Community unsupportive, disrespectful and devalue older adult contributions	[1, 5, 8, 11]
	Older people who are less well-off have access to public, voluntary & private services.	*Affordability*	Health Services	Costs of health services too high and causing financial hardship and concern, especially for those ageing-in-place	[1, 4, 11, 12]
			Housing	Lack of affordable housing creates a financial barrier, particularly for ageing-in-place	[2, 4, 11, 14]

			Public Transportation	High costs of public transport	[4, 11, 12]
	Unmatched				
	Older people are regularly consulted by public, voluntary & commercial services.				
	Service staff are helpful.				
	Older people are visible in the media & are depicted positively without stereo-typing.				

	Older people are included in community activities for 'families'.	
	Community-wide settings, activities & events attract all generations by accommodation age-specific needs.	
	Services & products suit varying needs & preferences provided by public & commercial services.	
	Schools provide learning about ageing & older people.	
	Facilitator	

Civic participation & employment	A range of flexible options for older volunteers are available.	*Social activities, participation & network*	Accessible, convenient & frequent social activities	Encourage contribution, meaningful involvement, social connections, & reduces isolation, particularly volunteering activities	[2, 4-6, 11, 12, 16, 19]
	Unmatched				
	Qualities of older employees are well-promoted.				
	Decision-making bodies in public, private & voluntary sectors encourage & facilitate memberships of older people.				

	A range of flexible & paid opportunities for older people to work are promoted.	
	Discrimination on the basis of age is forbidden in hiring, retention, promotion & training of employees.	
	Workplaces are adapted to the needs of disabled people	
	Self-employment options are promoted & supported for older people	
	Training in post-retirement options are provided for older workers.	

	Barriers				
Communication & information	Basic, effective communication system reaches residents of all ages.	*Accessibility*	Technological	Development of modern technology negatively impacts availability and accessibility of online information and availability of physical information	[3, 9, 11, 12]
			Information about services	Services and organisations offering activities do not communicate or signpost information	[4, 9, 11, 12]

	Regular & widespread distribution of information is assured, coordinated & central access is provided.		Technological	Development of modern technology negatively impacts availability and accessibility of online information and availability of physical information	[3, 9, 11, 12]
	Regular information of interest to older people is offered.		Information about services<br>Information about services	Services and organisations offering activities do not communicate or signpost information<br>Services and organisations offering activities do not	[4, 9, 11, 12]

				communicate or signpost information	
	Oral communication accessible to older people is promoted.		Technological	Development of modern technology negatively impacts availability and accessibility of online information and availability of physical information	[3, 9, 11, 12]
	People at risk of social isolation get one-to-one information from trusted individuals.		Information about services	Services and organisations offering activities do not communicate or signpost information	[4, 9, 11, 12]

	Printed information is accessible to all individuals.<br>Electronic equipment has large buttons & big lettering<br>Wide public access to computers, internet at no minimal charge in public places.		Technological	Development of modern technology negatively impacts availability and accessibility of online information and availability of physical information	[3, 9, 11, 12]
	Facilitators				
	Wide public access to computers, internet at no minimal charge in public places.	*Community Support*	Technological	Availability of computers helpful for accessing support & information	[11, 12]
	Unmatched				

	Public & commercial services provide friendly, person-to-person service on request.				
	Print & spoken communication uses simple & familiar words & straight-forward sentences.				
	Telephone services are accessible to all individuals.				
Community & Health services	***Barriers***				
	An adequate range of health & community support services are offered for	*Affordability*	Health Services	Costs of health services too high and causing financial hardship and	[1, 4, 11, 12]

	promoting, maintaining & restoring health.			concern, especially for those ageing-in-place	
	Health & Social services are conveniently located and accessible by means of transport	*Accessibility*	Physical access to amenities and facilities	1. Physical and disability-friendly accessibility to local buildings<br>2. Health Services<br>3. Community services<br>4. Shopping facilities<br>5. Senior centres<br>6. Activities & social gatherings	[1-6, 8, 9, 20]
		Transportation	Unsuitable public transport	Lack of public transport, physical accessibility to transportation and	[1, 3-6, 9, 11, 18]

			infrastructure, system & scheduling	unsuitable infrastructure a barrier to accessing services, facilities & social activities	
	Clear & accessible information is provide about health & social services to older adults.	*Accessibility*	Information about services	Services and organisations offering activities do not communicate or signpost information	[4, 9, 11, 12]
	Economic barriers impeding access to health & community services are minimised.	*Affordability*	Health Services	Costs of health services too high and causing financial hardship and concern, especially for those ageing-in-place	[1, 4, 11, 12]
	Facilitator				

	An adequate range of health & community support services are offered for promoting, maintaining & restoring health.	*Community Support*	Availability of services & facilities	Availability of shops, cafes & 'third places' such as libraries, particularly useful during winter and for social links	[4, 6, 9, 14, 18]
			Community-based programmes, neighbourhood associations and religious institutions	Encourages and provides support to older adults	[4-6, 14, 16]
			Intragenerational programmes and communities	Senior-specific programmes & community of people the same age	[2, 4, 11]

				promotes health, well-being & independence	
	Home care services include health & personal care & housekeeping.	*Housing*	Suitable housing and home services	Age-friendly homes, assisted living and home care services are beneficial and promote well-being	[4, 14]
	Residential care facilitates and older people's housing are located close to services & the community.	*Housing*	Close geographic proximity to community places & services	Close proximity to services, facilities and transportation services provides opportunities for community engagement, accessibility & reduces isolation	[1, 2, 4, 8]

	Voluntary services by people of all ages are encouraged & supported.	*Social activities, participation & network*	Accessible, convenient & frequent social activities	Encourage contribution, meaningful involvement, social connections, & reduces isolation, particularly volunteering activities	[2, 4-6, 11, 12, 16, 19]
	Unmatched				
	Delivery of services is coordinated & simple.				
	Staff are respectful, helpful & trained to serve older people.				
	Sufficient & accessible burial sites are available.				
	Community emergency planning takes into account				

	the vulnerabilities & capacities of older people.	

The role of urban environments in promoting active and healthy ageing: A systematic scoping review of citizen science approaches Appendices (1, 2 and 3)

Appendix 3

Table IV Citizen Science Appraisal Tool (CSAT) with full guidance for each question.

Section	Question	Y	N	?	Guidance
Science & Research	1) Is there a clear statement of the aims, objectives or goals of the study?				See CASP qualitative checklist[22] and cohort study checklist[23]
	2) Is it clear that the study used a citizen science approach?				***ECSA Principle 1*** - Citizens actively participate as contributors, collaborators, or study leaders to have a meaningful role in the study's scientific endeavour to generate new knowledge. Citizens may be involved in refining the study processes, materials and protocols. The main characteristics are (1) citizens are actively involved in research, partnership or collaboration with scientists or professionals; and (2) there is a genuine outcome, such as new scientific knowledge, conservation action or policy change".[24, 25]
	3) Is the degree of active engagement or participation of				**ECSA Principle 4 -** Citizens can engage and participate in multiple stages of the scientific/research process, which can include developing the research question or focus, designing the methods, data collection and analysis and

Leadership & Participation	citizens identified clearly by the study?				communicating the outcomes.[24, 26] Active engagement of citizen scientists in multiple stages of scientific/research process is preferred. Q) Has the study clearly identified its approach in terms of contributory (for the people), collaborative (with the people), or co-production (by the people)?.[27]
	4) Are the roles, responsibilities and type of partnership between citizens, scientists and stakeholders identified and transparent?				The roles and expectations should be made transparent, and citizens should be aware of their contribution to the research. Depending on the context of the study, it may be appropriate for citizens, scientists and stakeholders to have an equal partnership in the research. Q) Is the shift from participant to an active researcher made clear to citizens involved and has the study addressed this? [26]
Delivery & Data	5) Is the extent to which citizen scientists are actively engaged or collaborate in the data collection, analysis, and use/dissemination clear?				***ECSA Principle 4 –*** Citizens can engage and participate in multiple stages of the scientific/research process, which can include developing the research question or focus, designing the methods, data collection and analysis and communicating the outcomes.[24, 26] Q) Have citizens been engaged through a co-production or collaborative approach in the data collection, analysis and dissemination? During these processes, is there a clear partnership between citizen scientists with scientists and/or practitioners?[27, 28]

	6) Are citizen science data limitations or biases considered by the study?				***ECSA Principle 6*** - Citizen science data can contain bias or error, influence by factors such as variability among participants in relation to ability, commitment and effort. Accounting for this error and bias can mitigate this and can be addressed through well-developed protocols, appropriate and good design of activities or tasks that meet the study purpose, and good participant support.[24, 26] Multiple types of data and knowledge generation can be present in citizen science meaning studies should seek appropriate disciplinary standard which can include data quality and quality assurance standards, and peer-review of publications or any materials.[29]
Outcome, evaluation & open data	7) Are the main findings of the study clearly described?				See CASP qualitative checklist[22] and cohort study checklist.[23]
	8) Are the study's outcomes a direct result from the data-driven strategies and solutions generated by the citizen scientists?				A co-creation (by the people)[27] approach has been used and citizen scientists have been active collaborators throughout the study, which has ensured the relevance of the scientific endeavour and developed realistic outcomes or solutions.[24, 26, 30] Q) Have citizen scientists been fully engaged and empowered "not only as data collectors, but also as active collaborators"[28] in producing the strategies and outcomes of the study?

	9) Do the outcomes of the study have 'real world' decision making implications or impact?				**ECSA Principle 2** - Alongside answering a research question, outcomes such as informing actions, management decisions or policy are presented.[24] Q) Can the results be applied to the local population? Can the results be directly taken into real-world decision making? Is there a clear pathway to outcome and impact? [22, 31]
	10) Does the study report intention to track and/or tracking of long-term impacts, changes or 'ripple effects' of the study?				Q) Rather than decision making implications, has the study reported any long-term tracking of what has continued or happened after the study? This may include; (1) Impact of the study on citizen scientists that has led to ripple effects for these individuals or their community; (2) Sustainability of citizen science processes through ripple effects that have led to a continuation of community-engaged citizen science activities; (3) If outcomes or changes produced have led to long-term changes or impacts for citizens or their community after the study has finished; (4) The study reports a foundational partnership or longitudinal relationship with citizens with the intention to return and/or track impacts, changes or ripple effects.[28, 32-34] *Ripple effects are described further in Jagosh et al. [35]*
	11) Does the study report any evaluation of citizen knowledge, attitudes, actual and/or intended behaviours?				***ECSA Principle 3*** – Both scientists and citizens benefit from taking part, such as learning opportunities and personal enjoyment.[24] Evaluating participant knowledge can demonstrate if training and/or the project has been successful (in both content and skill) and can ensure sustained quality through participant understanding and engagement of tasks being completed correctly. Evaluating behaviour changes or intended behaviours may demonstrate intention to continue with CS activities.[31]

	12) Does the publication report any accessible dissemination plans or intentional mechanism for sharing the study and it's outcomes with citizens?				***ECSA Principle 5 –*** Clear communication or dissemination of the study and its outcomes are provided to citizens.[24] This may include how their data are being used, what the research, policy or societal outcomes are, or given the opportunity to 'see' their own data and its contribution (in suitable text and graphical forms).[26, 31]
	13) Are citizens invited to review or participate in the study's publication process?				Inviting citizens to participate or review the publication process will further strengthen the co-production and transparency of CS processes and dissemination. Q) Have studies reported any involvement of citizens in the publication process.
	14) Are the study's results and outcomes published in an open access format and/or shared in a publicly accessible format ?				***ECSA Principle 7 –*** Data from CS projects are publicly available and if possible, published in open access format.[24]
	15) Are citizen scientists acknowledged in the study's results and publications?				***ECSA Principle 8 –*** Citizens are acknowledged in project outcomes and publications.[24]

	16) Does the publication provide any critical evaluation of the study, methods and/or examination of its limitations?				**ECSA Principle 9-** Q) Is the study evaluated in any way for its scientific output, data quality, participant experience, wider societal impact, or policy impact? This may be to highlight trustworthiness, transparency or evaluation i.e. does the study report the citizen scientists evaluating the methods they have used and providing feedback?[24]

Scores will be categorised using the following scale system, adapted from Udani DM Checklist[36]:

**Low (0-6)**	**Low- Medium (7-12)**	**Medium (13-19)**	**Medium-High (20-26)**	**High (27-32)**

The role of urban environments in promoting active and healthy ageing: A systematic scoping review of citizen science approaches Appendices (1, 2 and 3)

References

1. Adorno G, Fields N, Cronley C, Parekh R, Magruder K. Ageing in a low-density urban city: transportation mobility as a social equity issue. *Ageing & Society*. 2018;38(2):296-320. doi:10.1017/S0144686X16000994

2. Fang ML, Woolrych R, Sixsmith J, Canham S, Battersby L, Sixsmith A. Place-making with older persons: Establishing sense-of-place through participatory community mapping workshops. *Social Science & Medicine*. 2016;168:223-229. doi:10.1016/j.socscimed.2016.07.007

3. Gustafsson S, Falk C, Tillman S, Holtz L, Lindahl L. Life filming as a means of participatory approach together with older community-dwelling persons regarding their local environment. *Scand J Occup Ther*. Sep 2018;25(5):347-357. doi:10.1080/11038128.2018.1502345

4. Novek S, Menec VH. Older adults' perceptions of age-friendly communities in Canada: a photovoice study. *Ageing & Society*. 2014;34(6):1052-1072. doi:10.1017/S0144686X1200150X

5. Parekh R, Maleku A, Fields N, Adorno G, Schuman D, Felderhoff B. Pathways to age-friendly communities in diverse urban neighborhoods: Do social capital and social cohesion matter? *Journal of gerontological social work*. 01 Jul 2018;61(5):492-512. doi:http://dx.doi.org/10.1080/01634372.2018.1454564

6. Mahmood A, Chaudhury H, Michael YL, Campo M, Hay K, Sarte A. A photovoice documentation of the role of neighborhood physical and social environments in older adults' physical activity in two metropolitan areas in North America. Empirical Study; Qualitative Study. *Social Science & Medicine*. Apr 2012;74(8):1180-1192. doi:http://dx.doi.org/10.1016/j.socscimed.2011.12.039

7. Tucket. Older Adults Using Our Voice Citizen Science to Create Change in Their Neighborhood Environment. *International Journal of Environmental Research and Public Health*. 2018;15(12):2685.

8. Ronzi S, Pope D, Orton L, Bruce N. Using photovoice methods to explore older people's perceptions of respect and social inclusion in cities: Opportunities, challenges and solutions. *SSM - Population Health*. 01 Dec 2016;2:732-744. doi:http://dx.doi.org/10.1016/j.ssmph.2016.09.004

9. Verma I, Huttunen H. Elderly-friendly neighborhoods. *Journal of Housing for the Elderly*. 2015;29:1-2.

10. von Faber M, Tavy Z, van der Pas S. Engaging Older People in Age-Friendly Cities through Participatory Video Design. *Int J Environ Res Public Health*. Dec 2 2020;17(23)doi:10.3390/ijerph17238977

11. Black K, Dobbs D, Young TL. Aging in community: mobilizing a new paradigm of older adults as a core social resource. *J Appl Gerontol*. Mar 2015;34(2):219-43. doi:10.1177/0733464812463984

12. Glover L, Dyson J, Cowdell F, Kinsey D. Healthy ageing in a deprived northern UK city: A co-creation study. *Health Soc Care Community*. May 22 2020;doi:10.1111/hsc.13036

13. Ronzi S, Orton L, Buckner S, Bruce N, Pope D. How is Respect and Social Inclusion Conceptualised by Older Adults in an Aspiring Age-Friendly City? A Photovoice Study in the North-West of England. *International Journal of Environmental Research and Public Health*. 2020;17(24):9246.

14. Buffel T, Phillipson C. Ageing in a Gentrifying Neighbourhood: Experiences of Community Change in Later Life. Article. *Sociology*. 2019;53(6):987-1004. doi:10.1177/0038038519836848

15. Salma J, Salami B. "We Are Like Any Other People, but We Don't Cry Much Because Nobody Listens": The Need to Strengthen Aging Policies and Service Provision for Minorities in Canada. *The Gerontologist*. 2020;60(2):279-290. doi:10.1093/geront/gnz184

16. Aw S, Koh G, Oh YJ, et al. Explaining the continuum of social participation among older adults in Singapore: From 'closed doors' to active ageing in multi-ethnic community settings. Empirical Study; Interview; Focus Group; Qualitative Study. *Journal of Aging Studies*. Aug 2017;42:46-55. doi:http://dx.doi.org/10.1016/j.jaging.2017.07.002

17. Barrie H, Soebarto V, Lange J, Mc Corry-Breen F, Walker L. Using Citizen Science to Explore Neighbourhood Influences on Ageing Well: Pilot Project. *Healthcare (Basel)*. Nov 1 2019;7(4)doi:10.3390/healthcare7040126

18. Garvin T, Nykiforuk CIJ, Johnson S. Can we get old here? Seniors' perceptions of seasonal constraints of neighbourhood built environments in a northern, winter city. Article. *Geografiska Annaler, Series B: Human Geography*. 2012;94(4):369-389. doi:10.1111/geob.12004

19. Hand CL, Rudman DL, Huot S, Gilliland JA, Pack RL. Toward Understanding Person--Place Transactions in Neighborhoods: A Qualitative-Participatory Geospatial Approach. *Gerontologist*. 2018;58(1):89-100. doi:10.1093/geront/gnx064

20. Tuckett. Older Adults Using Our Voice Citizen Science to Create Change in Their Neighborhood Environment. *International Journal of Environmental Research and Public Health*. 2018;15(12):2685.

21. Brookfield K, Scott I, Tinker A, Thompson CW. Perspectives on "novel" techniques for designing age-friendly homes and neighborhoods with older adults. *International Journal of Environmental Research and Public Health*. 2020;17(5)1800. doi:http://dx.doi.org/10.3390/ijerph17051800

22. Critical Appraisal Skills Programme. CASP Qualitative Checklist. Accessed 10/09/2020, https://casp-uk.net/wp-content/uploads/2018/01/CASP-Qualitative-Checklist-2018.pdf

23. Critical Appraisal Skills Programme. CASP Cohort Study Checklist. Accessed 10/09/2020, https://casp-uk.net/wp-content/uploads/2018/03/CASP-Cohort-Study-Checklist-2018_fillable_form.pdf

24. European Citizen Science Association. *Ten Principles of Citizen Science*. 2015. https://osf.io/ugy4t/

25. ECSA. What is Citizen Science? Accessed 10/09/2020, https://ecsa.citizen-science.net

26. Haklay M, Motion A, Balázs B, Kieslinger B, Greshake B T, Nold C, Dörler D, Fraisl D, Riemenschneider D, Florian H, Brounéus F, Hager G, Heuer K, Wagenknecht K, Vohland K, Shanley L, Deveaux L, Ceccaroni L, Weißpflug M, Gold M, Mazzonetto M, Mačiulienė M, Woods S, Luna S, Hecker S, Schaefer T, Woods T, and When T. . *ECSA's Characteristics of Citizen Science*. 2020.

27. King AC, Winter SJ, Chrisinger BW, Hua J, Banchoff AW. Maximizing the promise of citizen science to advance health and prevent disease. *Prev Med*. Feb 2019;119:44-47. doi:10.1016/j.ypmed.2018.12.016

28. King AC, Winter SJ, Sheats JL, et al. Leveraging Citizen Science and Information Technology for Population Physical Activity Promotion. *Translational Journal of the American College of Sports Medicine*. 2016;1(4):30-44. doi:10.1249/tjx.0000000000000003

29. Kosmala M, Wiggins A, Swanson A, Simmons B. Assessing data quality in citizen science. *Frontiers in Ecology and the Environment*. 2016;14(10):551-560. doi:https://doi.org/10.1002/fee.1436

30. Rowbotham S, McKinnon M, Leach J, Lamberts R, and Hawe P. . Does citizen science have the capacity to transform population health science. *Journal of Urban Health: Bulletin of the New York Academy of Medicine*. 2017;93(6):953-973.

31. Parrish JK, Burgess H, Weltzin JF, Fortson L, Wiggins A, Simmons B. Exposing the Science in Citizen Science: Fitness to Purpose and Intentional Design. *Integrative and Comparative Biology*. 2018;58(1):150-160. doi:10.1093/icb/icy032

32. King AC, King D K, Banchoff A, Solomonov S, Ben Natan O, Hua J, Gardiner P, Rosas L G, Espinosa P R, Winter S J, Sheats J, Salvo D, Aguilar-Farias N, Stathi A, Akira Hino A, Porter M M, and Our Voice Global Citizen Science Research Network. Employing Participatory Citizen Science Methods to Promote Age-Friendly Environments Worldwide. *Int J Environ Res Public Health*. Feb 27 2020;17(5)doi:10.3390/ijerph17051541

33. Moran M, Werner P, Doron I, HaGani N, Benvenisti Y, King A C, Winter S J, Sheats J L, Garber R, Motro H, et al. . Detecting inequalities in healthy and age-friendly environments: Examining the Stanford Healthy Neighborhood Discovery Tool in Israel. International Research Workshop on Inequalities in Health Promoting Environments: Physical Activity and Diet;. Israel: University of Haifa; 2015.

34. Winter SJ, Goldman Rosas L, Padilla Romero P, et al. Using Citizen Scientists to Gather, Analyze, and Disseminate Information About Neighborhood Features That Affect Active Living. *Journal of Immigrant and Minority Health*. 2016/10/01 2016;18(5):1126-1138. doi:10.1007/s10903-015-0241-x

35. Jagosh J, Bush PL, Salsberg J, et al. A realist evaluation of community-based participatory research: partnership synergy, trust building and related ripple effects. *BMC Public Health*. 2015/07/30 2015;15(1):725. doi:10.1186/s12889-015-1949-1

36. Wijewardhana UA, Meyer D, and Jayawardana M. Statistical models for the persistence of threatened birds using citizen science data: A systematic review'. *Global Ecology and Conservation*. 2020;21
