# Supplementary material for: The Role of Urban Environments in Promoting Active and Healthy Aging: A Systematic Scoping Review of Citizen Science Approaches
Source: J Urban Health. 2022 May 19;99(3):427–56. doi: 10.1007/s11524-022-00622-w (PMC9187804; doi:10.1007/s11524-022-00622-w)
Supplement: Supplementary file 2 — Supplementary file2 (PDF 56 KB) [file 11524_2022_622_MOESM2_ESM.pdf]

Supplementary Material 1

Table I An example of the keyword search and combination when searching Medline

**Database**	**Keyword search**
Medline	(((“Citizen Scien*”) *OR* (Participat*) *OR* (Collaborati*) *OR* (Engage*) *OR* (Partnership*) *OR* (“Community-based”) *OR* (Advocacy) *OR* (Photovoice) *OR* (Communit*) *OR* (“Citizen Engage*”) *OR* (CBPR) *OR* (“Community-Based Participatory Research”) *OR* (“Our Voice”) *OR* (Co-production) *OR* (“Participatory Action”) *OR* (“Public Participation”) *OR* (“Public Engagement”) *OR* (“Participatory Research”) OR (“Community Engage*”) *OR* (Community-led) OR (Co-production) OR (“Active Engage*”)) ***AND*** ((Urban*) *OR* (Built*) *OR* (“Urban Environment”) OR (“Built Environment”) OR (Outdoor*) *OR* (City) *OR* (Cities) *OR* (Age-Friendl*) *OR* (“Inclusive Communit*”) *OR* (“Physical Environment”) *OR* (Neighbourhood)) ***AND*** ((“Active Ageing”) *OR* (“Healthy Ageing”) *OR* (“Successful Ageing”) *OR* (“Ageing Well”) *OR* (“Positive Ageing”) *OR* (“Productive Ageing”) *OR* (“Meaningful Ageing”) *OR* (Ageing)) ***AND*** ((“Older Adult”) *OR* (Older) *OR* (“Older Woman”) *OR* (Older Women) *OR* (Older Man) *OR* (Older Men) *OR* (Senior) *OR* (Elder) OR (Community-dwelling))) kw, ti, ab.

The role of urban environments in promoting active and healthy ageing: A systematic scoping review of citizen science approaches Supplementary Material 1

1. Citizen Science Appraisal Tool Section Descriptors

(1) Science & Research

Section 1 identifies the aims, objectives, and/or goals of the study and clarifies that a CS approach has been used. This can demonstrate the validity and appropriateness of the research design and methods,[1, 2] and if studies have intentionally designed their approaches to demonstrate good quality CS. The presence of these aspects can strengthen CS and transition it towards being viewed as 'genuine' science in the traditional scientific community.[3]

(2) Leadership and Participation

Section 2 demonstrates the degree of active engagement of citizens within the study and the presence of a partnership between citizens and scientists, which are both principles presented by ECSA.[4, 5] Clear and planned engagement of citizens, with engagement of citizens throughout the entire process preferential, can demonstrate good quality.[3, 5] A transparent partnership and expectations can further strengthen the level of CS engagement, as it can shift citizens from 'participant' to 'active researcher'.[4]

(3) Data and delivery

Section 3 identifies studies who have fully engaged citizens in the data collection, analysis and dissemination processes. This level of active engagement is encouraged in CS approaches and demonstrates good quality CS.[4, 6] This section also identifies if studies have considered the quality and reliability of data, as well as any biases, errors or limitations that may be present, which is important for CS findings to be integrated and trusted within the scientific community.[7, 8]

(4) Outcomes, evaluation and open data

Section 4 identifies the level of CS engagement throughout the study processes, the presence of sustainable or 'real world' outcomes, critical evaluations of citizens or the study processes, and intentional mechanisms for disseminating outcomes, which are all indicators of good quality CS.[3-5] Fully engaging and empowering citizens, such as through co-production, aligns with ECSA CS values for preferred levels of engagement.[4, 5] The presence of 'real world' impacts or pathways can demonstrate sustainability of CS activities, such as through the continuation of community-engage CS activities,[6, 9-11] which are key for strengthening CS projects (Albert et al., 2021). Providing a critical evaluation of a study's processes can demonstrate quality, trustworthiness, and transparency,[4] and the evaluation of citizen knowledge or intended behaviours can indicate quality assurance of a project's delivery and ensuring participant understanding or learning.[3] Lastly, having accessible and open dissemination of outcomes is good practice [4] and can provide the opportunity for

citizens to 'see' their data, which can lead to long-term sustainability of CS studies.[3, 5]

Citizen Science Appraisal Tool Levels of Engagement

This review uses three different types of participation to guide the review provided by King et al. [12] These are:

1) 'For the people' – Contributory level of citizen science where citizens have limited engagement and are only involved to provide data (i.e. usually in the form of personal information or a biological sample). All other aspects of the research process are directed by the researchers.

2) 'With The People' - A type of collaborative citizen science where citizens actively and systematically collect data on a specific phenomenon (i.e. citizens involved in bird counts or online crowdsourcing). The data is then analysed, interpreted and disseminated by researchers and not citizen scientists.

3) 'By the people' – Produces a partnership or collaboration between citizen scientists and researchers in which citizens actively engage in the entire research process to drive and steer questions, objectives, collection, and interpretation of data, and developing and advocating outcomes and changes.

The role of urban environments in promoting active and healthy ageing: A systematic scoping review of citizen science approaches Supplementary Material 1

Studies can use the following methods, approaches or key words to describe this participation at any stage of design or methods to be included: (1) Citizen science (2) Citizen scientist/s or Citizen engagement; (3) Participatory (research, approaches, methods); (4) Participatory Action; (5) Collaborative/Collaboration; (6) Engagement; (7) Partnership; (8) Resident-engaged; (9) Community-Based Participatory Research (CBPR); (10) Advocacy/Advocate; (11) ‘Our Voice’; (12) Co-production; (13) For the people; (14) By the people; (15) With the people.

References

1. Critical Appraisal Skills Programme. CASP Qualitative Checklist. Accessed 10/09/2020, https://casp-uk.net/wp-content/uploads/2018/01/CASP-Qualitative-Checklist-2018.pdf

2. Leung L. Validity, reliability, and generalizability in qualitative research. *J Family Med Prim Care*. Jul-Sep 2015;4(3):324-327. doi:10.4103/2249-4863.161306

3. Parrish JK, Burgess H, Weltzin JF, Fortson L, Wiggins A, Simmons B. Exposing the Science in Citizen Science: Fitness to Purpose and Intentional Design. *Integrative and Comparative Biology*. 2018;58(1):150-160. doi:10.1093/icb/icy032

4. European Citizen Science Association. *Ten Principles of Citizen Science*. 2015. https://osf.io/ugy4t/

5. Haklay M, Motion A, Balázs B, Kieslinger B, Greshake B T, Nold C, Dörler D, Fraisl D, Riemenschneider D, Florian H, Brounéus F, Hager G, Heuer K, Wagenknecht K, Vohland K, Shanley L, Deveaux L, Ceccaroni L, Weißpflug M, Gold M, Mazzonetto M, Mačiulienė M, Woods S, Luna S, Hecker S, Schaefer T, Woods T, and When T. . *ECSA's Characteristics of Citizen Science*. 2020.

6. King AC, Winter SJ, Sheats JL, et al. Leveraging Citizen Science and Information Technology for Population Physical Activity Promotion. *Translational Journal of the American College of Sports Medicine*. 2016;1(4):30-44. doi:10.1249/tjx.0000000000000003

7. Kosmala M, Wiggins A, Swanson A, Simmons B. Assessing data quality in citizen science. *Frontiers in Ecology and the Environment*. 2016;14(10):551-560. doi:https://doi.org/10.1002/fee.1436

8. Williams J, Chapman, C., Leibovici, D. G., Loïs, G., Matheus, A., Oggioni, A., Schade, S., See, L. and van Genuchten, P, P. L. . Maximising the impact and reuse of citizen science data In: Hecker S, Haklay, M., Bowser, A., Makuch, Z., Vogel, J. and Bonn, A., ed. *Citizen Science: Innovation in Open Science, Society and Policy* UCL Press; 2018:321-336.

9. King AC, King D K, Banchoff A, Solomonov S, Ben Natan O, Hua J, Gardiner P, Rosas L G, Espinosa P R, Winter S J, Sheats J, Salvo D, Aguilar-Farias N, Stathi

A, Akira Hino A, Porter M M, and Our Voice Global Citizen Science Research Network. Employing Participatory Citizen Science Methods to Promote Age-Friendly Environments Worldwide. *Int J Environ Res Public Health*. Feb 27 2020;17(5)doi:10.3390/ijerph17051541

10. Moran M, Werner P, Doron I, HaGani N, Benvenisti Y, King A C, Winter S J, Sheats J L, Garber R, Motro H, et al. . Detecting inequalities in healthy and age-friendly environments: Examining the Stanford Healthy Neighborhood Discovery Tool in Israel. International Research Workshop on Inequalities in Health Promoting Environments: Physical Activity and Diet;. Israel: University of Haifa; 2015.

11. Winter SJ, Buman MP, Sheats JL, et al. Harnessing the potential of older adults to measure and modify their environments: long-term successes of the Neighborhood Eating and Activity Advocacy Team (NEAAT) Study. *Transl Behav Med*. Jun 2014;4(2):226-7. doi:10.1007/s13142-014-0264-1

12. King AC, Winter SJ, Chrisinger BW, Hua J, Banchoff AW. Maximizing the promise of citizen science to advance health and prevent disease. *Prev Med*. Feb 2019;119:44-47. doi:10.1016/j.ypmed.2018.12.016
