# Supplementary material for: The Role of Urban Environments in Promoting Active and Healthy Aging: A Systematic Scoping Review of Citizen Science Approaches
Source: J Urban Health. 2022 May 19;99(3):427–56. doi: 10.1007/s11524-022-00622-w (PMC9187804; doi:10.1007/s11524-022-00622-w)
Supplement: Supplementary file 3 — Supplementary file3 (PDF 392 KB) [file 11524_2022_622_MOESM3_ESM.pdf]

Supplementary Material 2

Table I Detailed Overview of the Critical appraisal Outcomes of 23 included studies

Author, Year	Question	Answer	Reason	Point
Barrie et al. (2019)	1	Yes	This pilot study aimed to test a new smart phone-based audit tool using an innovative methodology—citizen science—in order to explore how and why older people engage with public green spaces.	2
	2	Yes	A citizen science approach with a co-created model was used to evaluated public green spaces not based on researchers valued judgement	2
	3	Yes	Co-created whereas citizen scientists, older people not only collected data but were also engaged in preliminary analysis of the data and, most importantly, contributed feedback and ideas on the methods, process, audit tool and the design of the proposed larger project.	2
	4	Yes	Clear that citizens have the role of being trained as citizen scientists, collect data using the data tool and preliminary analyse of data and feedback of the tool.	2

	5	Yes	Citizen Scientists were engaged in collecting data in their own chosen locations using an online tool (or paper-based tool) and were engaged in the interpretation of this data through interviews and pre-liminary analysis. No dissemination was discussed by the paper.	2
	6	Yes	This pilot engaged a small, self-selected group of adults interested in participating in citizen science and may not represent the general older population. Further work needs to be done with wider groups of older adults, including those with reduced mobility, greater frailty and/or poorer health, and from different cultural backgrounds to test both the potential and reliability of the audit tool.	2
	7	Yes	Broken down into use of tool, audit of data and participant reflection on senior citizen science.	2
	8	Unclear	The main findings of the project are based on the data collected by citizen scientists using the online/paper tool in their chosen locations and provided an understanding of key design elements for these individuals. However, it was unclear if they were actively engaged in the development of the project and no solutions provided to this but instead the focus was on evaluating the citizen scientists' experiences in the study and the use of the tool.	1
	9	Yes	The findings provide a pathway to real-world decision making in terms of the design elements citizens highlighted in green spaces, alongside information of technology and becoming comfortable with it.	2

	10	Unclear	Although the article says ‘Participants showed a keenness to be further engaged with future citizen science projects beyond just data collection, indicating that whenever possible they would like to be involved in all stages of future research projects’ which shows a sustainable element of the citizen science process, it does not report any ‘ripple-effects’ or highlight any projects participants have been involved in since.	1
	11	Yes	Citizens were involved in evaluating the tool and citizen science approach.	2
	12	No	None reported.	0
	13	No	None reported.	0
	14	Yes	Open access	2
	15	Yes	Participants acknowledged in acknowledgements section.	2
	16	Yes	Citizens evaluated the project and the tool experience which were included in results and discussion.	2
**Total Score = 26 (Medium-High)**				
Mahmood et al. (2012)	1	Yes	The purpose of this study was to conduct a participatory research process with community-dwelling older adults using photovoice method to identify neighbourhood physical environmental features and social aspects that influence physical activity in older adults.	2

	2	Yes	Participatory research & participatory method called photovoice was used where citizens are actively involved.	2
	3	Yes	The study clearly reports and identifies the level of active engagement from citizens throughout the initial session, data collection and discussion of findings. – 'In the initial session, the participatory nature of photovoice method was emphasized and training was used to identify three important reasons for engaging community. For the photovoice activity, participants photographed physical and social aspects that they perceived as barriers to physical activity behaviour, and they had two weeks to do so and mail back the camera. Photos were printed and participants were asked to select 6-8 photographs that best reflected the issue trying to be captured. Participants also attended a discussion session where participants discussed and reflected on their photos and was facilitated by the researcher who summarised the group findings. Key issues and recommendations were generated at the discussion by participants. The final dataset was analysed by the researchers.'	2
	4	Yes	In the initial session, it is clear that the engagement element was communicated to participants and their roles were clear in collecting data and discussing it to create recommendations.	2
	5	Yes	Participants were directly engaged in the photovoice data collection and used this to highlighted facilitators or barriers in their neighbourhoods. Participants were also engaged in selecting the photos that reflect the issues they were trying to highlight, as well as discussing the pictures in group discussions with other participants to produce recommendations. However, it is also clear that researchers undertook the overall data analysis, and no dissemination of the findings were reported.	2

	6	Yes	The study considers it limitations - 'The findings were not analysed in relation to any information on the participants' actual physical activity. However, the participants included individuals with variability in individual- and neighbourhood characteristics and the consistency of the findings from the two cities suggests that the barriers and facilitators were real and important. Data were collected in only eight neighbourhoods… potentially limiting the generalizability of our results'	2
	7	Yes	Clear main outcomes of the project are described in terms of the barriers and facilitators.	2
	8	Yes	The seven themes of the study (outcomes and recommendations) are directly generated by the photovoice methods and groups discussions that participants were actively engaged in.	2
	9	Yes	Seven clear themes that can be included in altering urban environments as well as information about peer support and community-based groups.	2
	10	Unclear	The study reports 'Several participants expressed the desire to form a seniors' coalition to create change in their neighbours using their photographs and joining with others from the study. In order to successfully create these senior advocate groups, more resources are needed to continue the project on the local level.' This shows an approach to sustainable citizen science but does not report any further actions or impacts on citizens or their local community.	1
	11	No	None reported.	0

	12	No	None reported.	0
	13	No	None reported.	0
	14	No	Not open access.	0
	15	Yes	Participants acknowledged in acknowledgements- ‘The authors are grateful to the older adult research participants in Vancouver and Portland metropolitan areas for their enthusiasm, efforts, and time.’	2
	16	No	None reported.	0
**Total Score = 21 (Medium-High)**				
Adorno et al. (2018)	1	Yes	The purpose of this article is to present findings about transportation mobility using a social justice and social equity lens. We focus on the perceptions and experiences of older adults regarding the meaning of transportation to them as they age in their current environment.	2
	2	Yes	Uses 3 older adults as peer researcher /community liaison (with a researcher, for some interviews) involved in: planning, recruitment, data collection and analysis phases and described as community-based methods.	2
	3	Yes	Yes 3 older adults (not all participant) involved in: planning, recruitment, data collection and analysis phases	1
	4	Yes	It is clear that citizens will be involved in peer research, interviewing, translation.	2

	5	Yes	Yes – peer research, interviewing, translation	2
	6	Yes	Strategies were described to address bias in the data analysis and limitations were considered i.e. “iterative, consensus coding process to identify researcher bias and continuous clarification about their meaning with participants during interviews and focus groups”; “member checks with study participants and the community liaison to obtain feedback on the preliminary themes, to establish trustworthiness of the data and to increase the credibility of the findings” also acknowledge selection bias and lack of LGBT participants	2
	7	Yes	Broken down into the different themes that were clear and related to the study focus. Qualitative verbatim quotes provided, covering a range of scales/locations.	2
	8	Unclear	Although 3 older adults and community liaisons were present in the planning, recruitment, data collection and analysis phases, it is unclear their level of active engagement in the interviews/focus groups and guides used for this, as well as the direct engagement of all other older adults involved in the project.	1
	9	Yes	Implications for pathway for decision making rather than impact: for WHO liveable communities initiative 2007a, for understanding older people’s transportation mobility and challenges, issues of social equity, need for collective action, past policy failures	2

	10	No	None reported.	0
	11	No	None reported.	0
	12	No	None reported.	0
	13	No	None reported.	0
	14	No	Not open access	0
	15	No	No acknowledgement.	0
	16	Unclear	Evaluation was completed in the sense of considering the value of citizen engagement for supporting urban change, but in no other way.	1
**Total Score = 17 (Medium)**				
Annear et al. (2014)	1	Yes	(For the four stages as well as for the stage described in this paper)- To develop recommendations for urban environmental change following a natural disaster, based upon the findings of three research phases, and disseminate to key stakeholders.	2
	2	Yes	Described as a participatory investigation and collaborative process with older adults and has involved including older adults in photovoice investigation and focus group with older adults and 8 older adult advisors to create and critique solutions.	2

			Described as a collaborative process and participatory investigation - “rationale for involving older adults in the research process is the imperative to inform change and connect community members with more powerful actors”	
	3	Yes	Described as a collaborative process with older adults where older adults (including 8 older adult advisors who provided guidance) create and critique solutions based on data collected during the photovoice stage. - ‘with the people’ collaborative research, PAR method, not explicitly identified as citizen science	2
	4	Yes	Older adult advisors provide guidance and critique to the research process and results and older adults were engaged in the focus group stages to create and critique solutions.	2
	5	Yes	The first two stages of data collection were driven by researchers to build an understanding of the geographical area and where older adults resides. The last two stages were driven by participants who were engaged in activity diaries and photovoice procedures to collect data and group discussions with other participants to create and critique solutions for rebuilding the city. Participants were also engaged in the data analysis and were presented themes that were used in discussions to create recommendations and a proposed timeframe.	2
	6	Yes	Considered but not controlled for: Reference/advisory group from varying socio-economic backgrounds high level of education, not intended to be representative	2

	7	Yes	Included barriers and recommendations produced by older adults	2
	8	Yes	Participants were directly engaged in collecting data, analysing the data, and using this to produce recommendations and solutions as well as time frames. Emergent themes with examples from empirical data; specific and detailed recommendations	2
	9	Yes	Although there's no concrete link expressed with policy makers with decision making powers, although some members of advisory group were volunteers in the Elder Care Canterbury Consumer Group, which "report problems and issues to the Canterbury District Health Board and other agencies", there are pathways present with the findings and citizens to producing real world decision making.	2
	10	No	None reported.	0
	11	No	No evaluation of the participatory methods or citizens knowledge is documented	0
	12	Yes	the participatory phase described in this paper addresses this and gives participants opportunity to make recommendations based on research interpretations of data	2
	13	No	None reported.	0
	14	No	Not open access.	0

	15	Yes	Acknowledged in the acknowledgements section.	2
	16	No	None reported.	0
**Total =22 (medium-high)**				
Aw et al. (2017)	1	Yes	This study aims to identify and explain the continuum in which older people in Singapore participate in community and social life, highlighting the influence of culture and policy context on social participation.	2
	2	Yes	Described as three qualitative methods that integrated ethnographic approaches with methodological approaches of community-based participatory research. These methods included photovoice documentation and discussion groups , (b) Walking Through Spaces or ‘go-along’ inter- views and (c) Community Focus Groups Discussions which tapped into participatory learning for action (PLA) exercises. Citizens are actively engaged in the methods. The study describes a structural ethnographic approach in order to capture social behaviours of older adults in the community, and the influence of cultural and retirement context. It describes itself as using methodological approaches of CBPR in order to allow participants to interact with their community to record information and to allow the research to observe the community through the eyes of participants.	2
	3	Yes	Participants are engaged in the three methodological stages(photovoice, ‘go-along’ interviews, group discussions) to focus on topics relating to participation in community activities, and other ways of social participation and expand these perspectives	2

			using photovoice by asking elder participants to photo-document for one month, anything they felt showcased ‘lives of elders’ in Whampoa.	
	4	Yes	Participants are engaged with the researchers throughout the methodological stages in their community to show how they participate in community and social life	2
	5	Yes	Methodological approaches of community-based participatory research are used which actively engage citizens in data collection. However they are not engaged in any other stages of the research such as solution building or data analysis.	2
	6	Yes	Appropriate and good design of activities or tasks have been used to meet the study purpose (i.e. We also used a neighbour-level ethnographic and participatory approach, combining three qualitative methods in such a way to focus, expand then compare perspectives for triangulation) and they identify a limitation of not managing to interview the more vulnerable older adults, though we nonetheless tried to ac- count for their perspectives through older adults who went around speaking to them in Photovoice, and community leaders who worked directly with these older adults.	2
	7	Yes	Five themes are shown to emerge from the data collected by citizens that highlight how they participate in their community and social life.	2
	8	Unclear	Although the outcomes are achieved through a CBPR methods that engaged citizens, they were not actively engaged in other aspects such as building solutions or data analysis.	1

	9	Yes	The study highlights how their findings prove a pathway towards cultural and policy implications and how this could be achieved.	2
	10	No	None reported.	0
	11	No	None reported.	0
	12	No	None reported.	0
	13	No	None reported.	0
	14	Yes	None reported.	2
	15	No	None reported.	0
	16	No	None reported.	0
**Total Score = 19 (Medium)**				
	1	Yes	This study aims to advance our understanding of older adults' perceptions and the broader contributions of community residents in affecting dignity and independence in everyday interactions with older adults.	2

Black et al., 2015	2	Yes	"Using participatory action research, multiple methods of qualitative inquiry, and tenets of appreciative inquiry, this article reports on a community-based initiative aimed to better understand the positive aspects of aging with dignity and independence". Described as "The process is collaborative and equitable in that partners are included throughout the research, from study design, data collection, analysis, and dissemination". This included community forums, focus groups and online surveys. The tools used were endorsed by the community advisory group. PAR and multiple methods of qualitative inquiry were utilized to gain an in- depth understanding of how older adults, and the broader community of persons of all ages, view and promote aging with dignity and independence in the context of everyday life. PAR is a systematic approach to inquiry in which those affected by an issue under study are included in the research for the purpose of social action. Described as "The process is collaborative and equitable in that partners are included throughout the research, from study design, data collection, analysis, and dissemination".	2
	3	Yes	An advisory group was comprised of 20 community members that leveraged key community-based providers, volunteers, experts, and stakeholders in aging (one third of this were older adults) and the project was discussed with this group throughout five meetings during the project duration. Data collection tool used for community forums were based on participants providing a narrative based on 'tell your story'. The focus group and open-ended surveys were developed to elicit older adults' perceptions and followed a semi-structured interview guide but allowed participants to expand further on points. The interview guide was pretested with older adults. Data were shared with older adults who participated in the interviews as	2

			a source of member checking to enhance trustworthiness of the data and with the advisory group throughout the ongoing analysis.	
	4	Yes	Individuals and older adults are recruited for advisory groups to discuss and direct the project, collect data, and be engaged in the data analysis.	2
	5	Yes	The advisory group (which includes older adults and community members) are actively engaged in directing the project, older adults are engaged in the two stages of data collection, and the data they provide in the community forums and focus groups are member checked by them. The data analysis is also shared with the advisory group.	2
	6	Yes	The credibility of the data was enhanced through a number of practices: (1) triangulation of data occurred by maintaining and recording events throughout data collection and analysis; (2) the use of a multidisciplinary research team (social gerontology, social work, and graduate students from different social behavioural and aging disciplines) involved in the data analysis and interpretation of themes; (3) multiple researchers analysed the data independently; (4) researchers reviewed the findings at different stages of analysis; (5) the process of data collection was enhanced by careful documentation and organized record keeping so that data were maintained in retrievable and easily audited form by peers (Miller & Crabtree, 1999); (6) multiple modalities of qualitative methodologies; and (7) data were shared with older adults who participated in the interviews as a source of member checking to enhance trustworthiness of the data.	2

	7	Yes	8 clear actionable themes are produced in the findings	2
	8	Yes	The project outcomes are co-created with community members and older adults, and developed through collaboration with these individuals collecting, checking, and analysing the data.	2
	9	Yes	The outcomes highlight pathways for micro and macro-level community-based actions and real-world decision-making impacts.	2
	10	No	None reported.	0
	11	No	None reported.	0
	12	No	None reported.	2
	13	No	None reported.	0
	14	Yes	Open Access.	2
	15	No	None reported.	0
	16	No	None reported.	0

**Total Score = 22 (Medium-High)**				
Brookfield et al. (2020	1	Yes	"We draw on of the eight techniques as they were employed at various participatory design events, as well as on feedback collected from the older adults (typically over 60 years old) who participated in these events, to reflect on the value each might have as a mechanism for including older people in environmental design decisions."	2
	2	Yes	Citizens were engaged in multiple approaches and stages of the methods which were called co-design workshops. "We organized a series of participatory design events across the United Kingdom (UK) over a three-year period. These included three co-design workshops, three design review events, and 22 one-to-one interactions with diverse older adults. Within these, we implemented a collection of more "traditional" techniques, including structured interviews and focus groups, as well as more "novel" engagement techniques. For example, at the co-design workshops held in central Manchester and Hackney Wick, we combined the techniques of walking interviews and photovoice in a single "Walk and Talk" activity. This meant that, at each location, participants took one walk around the neighbourhood in which we worked and, on this walk, completed a walking interview and, addressing the concerns of photovoice, took photographs of the environments traversed and then discussed these photographs at the end of the walk. The study used co-design workshops in which there were 'novel' engagement techniques used. These included photovoice, walking interviews, photo-elicitation, town hall meetings, talking mats, model-making, participatory mapping, drawing, design fair which were all participatory design events across the UK. These techniques engaged older adults within the methods of the study.	2

	3	Yes	Throughout the methods of the study, the ‘novel’ engagement techniques were clearly described and how citizens were engaged at each stage as well as providing feedback on these engaged and integrated activities. ‘With the people’ but not by the people are citizens were also observed and there is no clear explanation on the analysis of the results.	2
	4	Yes	It is clear that citizens were engaged in the methods and providing feedback whereas researchers organised the events and observed participants	2
	5	Unclear	Although it is clear how the citizens were actively engaged in the methods of this study and providing feedback, it is unclear on how or if they were engaged in the analysis section (as this is not reported) and the dissemination of outcomes and feedback provided.	1
	6	Yes	The study discusses and considers their limitations but not focused on data quality/bias - *Limitations* of our work include the relatively small numbers of participants engaged in the co-design events and one-to-one interactions, and the relatively small (although diverse) number of contexts within which the engagement techniques were trialled. Future iterations of the work could usefully implement a set of novel engagement techniques in a wider range of contexts with a larger number of diverse older adults. This could support firmer conclusions on the potential for more novel forms of engagement to facilitate the involvement of diverse older adults in environmental design decisions.	2

	7	Yes	The outcomes provide a clear reflection and overview of feedback on each of the engagement methods used at the co-design workshops.	2
	8	Unclear	It is unclear how much the strategies and solutions are a direct result for citizen data-driven strategies. Citizens were actively engaged in the methods of this approach and providing feedback on the methods. However, they were not actively engaged or collaborating at any other stage, so the outcomes are a result of their engagement in the methods but not through any other strategies or solutions.	1
	9	Yes	The results of this study of how these engagement techniques could be used with older adults (based on their own feedback) could be applied to further engaging these individuals in environmental and community concerns and decisions and could provide a pathway to engaging these individuals in informing these decisions.	2
	10	No	None reported.	0
	11	No	Although citizens perspectives were asked for feedback, citizens were not tested or evaluated on their knowledge of the tools use or their implementations etc.	0
	12	No	None reported.	0
	13	No	None reported.	0

	14	Yes	Open Access.	2
	15	Yes	The authors thank the reviewers and the older adults and design students who participated in this element of the project.	2
	16	Unclear	Citizens provided feedback on the tool but unclear on the level of evaluation.	1
**Total Score = 21 (medium-high)**				
Buffel (2018)	1	Yes	The purpose of this article is to provide insights into the process of co-producing a research project with older residents living in low-income neighbourhoods in Manchester, United Kingdom. The project was unique in involving and training eighteen older people as co- researchers who took a leading role in all phases of a study aimed at developing "age-friendly" communities.	2
	2	Yes	Yes, older adult citizens are co-researchers in a study aimed at developing the "age-friendliness" of their communities. The project is co-led by older adults and includes their experiences of older peoples as they step beyond the traditional role of consultee to that of interviewer, researcher, exploring the co-researchers, motivation for participant in the project. They are engaged in multiple stages of the study as co-researchers and co-designers.	2
	3	Yes	Citizens are actively engaged through the study - This research is by the people where co-researchers participate in co-designing the research objective, co-producing research materials, collecting data, analysing data, co-producing findings,	2

			training sessions, reflection meetings, dissemination workshops. However, the other older adults engaged in the process are only engaged in providing data in the focus group and interviews.	
	4	Yes	Older adults are clearly identified as co-researchers and are part of workshops that aimed to agree on roles and responsibilities and develop a peer support network.	2
	5	Yes	Older adults are clearly engaged in a 'by the people' approach where they are co-researchers throughout and actively engage in the data collection analysis and dissemination. However, the other older adults engaged in the process are only engaged in providing data in the focus group and interviews, demonstrating a 'with the people' approach.	2
	6	Unclear	The study identifies challenges that were outcomes of the co-production methods used such as developing collaborative partnerships and negotiating power relationships, as well as projects creating a divide between privileged and disadvantaged groups. However they do not discuss limitations of their study, population used or data quality.	1
	7	Yes	The outcomes of the study demonstrate the experiences and motivations behind becoming a co-researcher for older adults, including motivation, community engagement, personal development and understanding, relationship between co-researcher and interviewees. The outcomes also highlight opportunities and challenges in developing work around co-production with older people.	2

	8	Yes	Older adults were engaged throughout the entire process so that outcomes are a direct result from the questions and data they co-produced and generated.	0
	9	Unclear	Although the results demonstrated the experiences, motivations and skills undertaking co-research, these were based on those specifically of older adults in Manchester and may differ for other older adults in different age & ethnicity groups, locations etc. So it may provide a pathway to have implications for further research and informing actions from co-production research, but it is unclear.	1
	10	Yes	The study reports that the co-researchers have now formed a permanent group and are applying for funding for age-friendly activities. They also reported that further evaluation and research will be needed to track the impact of the group.	2
	11	Unclear	Reflection meetings were held to understand co-researcher’s involvement and motivation but there was no evaluation of the study methods or citizen knowledge etc.	1
	12	No	Although the results are disseminated with stakeholders and action plans fed into Manchester age-friendly program, the study does not report dissemination plans or sharing the outcomes of findings with citizens.	0
	13	No	None reported.	0
	14	No	None reported.	0

	15	Yes	The author would like to express gratitude to the School of Social Sciences at the University of Manchester; the Age-Friendly Manchester team at the City Council, Chorlton Good Neighbours, Whalley Range Community Forum, the Manchester Institute for Collaborative Research on Aging and all of the co-researchers and participants in the study.	2
	16	Unclear	The study reports 'a strategy for evaluating the impact of the initiatives and strengthening the links between University and co-researchers was also developed' but no further information was reported on this.	1
**Total Score = 20 (Medium-High)**				
Buffel & Phillipson(2019)	1	Yes	This article explores the experiences of older people living in an urban neighbourhood undergoing residential and commercial gentrification.	2
	2	Yes	The research in this article derives from a study which aimed to train older people as co-researchers in developing 'age-friendly communities; the project used a participatory action research (PAR) framework, centred around three principles: participation; collaboration; and community action. A 'co-research' approach was adopted to allow older people's active participation as *partners* in the research process.	2
	3	Yes	Older people took a leading role both in developing and implementing the study as well as translating findings into policy recommendations and action; Eighteen people (10 females and eight males), aged between 58 and 74 years, were recruited and trained as co-researchers who collaborated on the development of the research design. However, the other older adults engaged in the process are only engaged in providing data in the focus group and interviews.	2

	4	Yes	Citizen scientists were co-researchers and trained/took part in reflecting meetings to address relevant stages of the research cycle (from design to collecting, analysing, and disseminating findings).	2
	5	Yes	Yes, it is clear that citizens are co-researchers and engaged in all stages (through a ‘by the people’ approach) in collection analysing and disseminating data.	2
	6	Yes	Although this study identifies important dimensions to the experiences of older people in a gentrifying neighbourhood, the research also has some limitations (study highlights three limitations of location, PAR methodology limitations, underrepresenting particular groups in the community. Study considers methodology limitations.	2
	7	Yes	The outcomes and findings of the study are themes that are clearly described and meet the focus of the paper.	2
	8	Yes	Citizens were trained in collecting data and undertook data collection, analysis, and the choice of quotes for the findings. They were directly engaged and empowered in these stages as co-researchers.	2
	9	Yes	‘Studying the lives of older people in gentrifying areas contributes a great deal both to the broad picture about the impact of urban change, and about the various ways in which people develop strategies for managing their lives’- the study provided an overview of experiences of older adults and urban change and highlight factors (such as cost of housing, changes in types of shops etc) and the impact of this that could provide a pathway to real life decision-making for these individuals in this area related to urban change.	2

	10	Yes	Following their involvement in the study, the co- researchers developed as 'ambassadors' for the co-production model, promoting the age-friendly approach through activities such as: contributing to the development of policies within the local authority; speaking at (inter)national conferences; participating in new research projects; and contributing to change within the community, including the restoration of a much-valued bus service within one of the neighbourhoods.	2
	11	No	None reported.	0
	12	No	None reported.	0
	13	No	None reported.	0
	14	Yes	Open Access.	2
	15	Yes	Acknowledged in the Acknowledgements section	2
	16	No	None reported.	0
**Total Score = 24 (Medium-High)**				
Chui et al. (2020)	1	Yes	This study examined the effects of using photo-voice as an alternative method in facilitating older adults' civic participation in Hong Kong; In this study, we aimed to explore how the process of participating in a photo-voice project facilitated participants' civic participation and propose an empowerment-based participatory photo-voice training model.	2

	2	Yes	This study uses focus groups to evaluate citizens scientist's experiences who previously took part in an empowerment-based participatory photo-voice training, take home assignment and exhibitions. In this study, older adults took part in group interviews and were asked questions and discuss their feelings and opinions about the photovoice experience they were previously involved in and trained in.	2
	3	Unclear	Citizens shared their feelings and opinions in the group interviews about their experience and were actively involved in the photo voice stage (but this was a previous study and not the aim of this study) but were not involved in the focus group preparation or data analysis/dissemination.	1
	4	Yes	Citizens were re-invited back to discuss their experiences in group interviews.	2
	5	Unclear	Citizens were actively engaged in sharing their feelings and opinions in the group interviews about their experience (as well as actively engaged in the previous study) but they were not engaged in the analysis or dissemination of outcomes.	1
	6	Yes	Limitations are addressed in this study, including 'findings may not be directly transferable to other older adults' and 'second, because participation in this photo-voice project was entirely voluntary, older adults who are willing to participate may already have been the more "active" group, thus creating a self- selection effect'	2
	7	Yes	The views and opinions of citizens and their experiences of using photovoice and being empowered are clearly described by the study.	2

	8	No	Citizens were not fully engaged and empowered in this study but were instead invited back to discuss their previous experiences.	0
	9	Unclear	The outcomes demonstrate the value of photo-voice in this context and engaging older adults in research to provide a relevant pathway for decision making, but the outcomes relate more to the use of photovoice for developing further studies/research and engagement with older adults.	1
	10	No	None reported.	0
	11	Yes	Citizens were evaluated in this study on their training and use oh photo-voice and how it can convey older adults' views, broaden perspectives, and enables knowledge acquisition and dissemination. It evaluated their knowledge and attitudes towards photovoice.	2
	12	No	None reported.	0
	13	No	None reported.	0
	14	No	Not Open Access.	0
	15	No	None reported.	0

	16	Yes	The study does critically evaluate the use of photo-voice as an empowerment approach for older adults and received feedback from citizens about the use of this method	2
**Total Score = 17 (Medium)**				
Fang et al. (2016)	1	Yes	(1)This paper illustrates how applications of community-based participatory research methods, in particular, participatory community mapping workshops (PCMWs),can be used to access experiences of place, identify facilitators and barriers to accessing the built environment and co-create place-based solutions among older people and service providers in a new affordable housing development in Western Canada;	2
	2	Yes	Participatory Community Mapping Workshops were run where citizens directed group walks and mapping workshops for generating ideas and solutions (with each workshop building on the previous). Citizens directed the mapping process and were co-owners of the maps produced, as well as building relationships with service providers and researchers in the workshops to develop achievable goals and actions. The approach used is described as a community-based participatory research method (participatory mapping research). Citizens are described as those who direct the workshop processes, outcomes, and solutions as a way to further understanding sense-of-place and experiences among older adults.	2

	3	Yes	Citizens are engaged in the CBPR methods through walks and co-owning the mapping processes. They were also engaged in earlier research where strong relationships and community ties were developed prior to the PCMWs. It is clear that citizens are actively engaged in the four workshops and co-conducted the data analysis for workshops 2 and 3.	2
	4	Yes	The aims of the workshops identify the clear focus of each workshop and how citizens can direct and co-own the stages. Citizens were also engaged as co-owners of maps and co-conductors of data analysis.	2
	5	Yes	Citizens are engaged as generators, co-owners and co-conductors of the data and data analysis as well as building actions and solutions with other workshop members/stakeholders.	2
	6	Unclear	Limitations of the study are considered in the form of (1) Not video recording the workshops to understand how maps are drawn; (2) the resource intensive and time-consuming factors or participatory methods and the study faces a challenge of maintaining long term impact and outcomes. However, there is no consideration of data quality or bias that may have been impacted by the methods.	1
	7	Yes	The key findings from the workshops are described clearly and separated into different categories.	2
	8	Yes	Citizens are active collaborators and drive the workshops as well as the action points and solutions that are generated	2

	9	Yes	The outcomes highlight the experiences of older adults and their needs including the services, activities and solutions that are wanted by older adults in their community which could provide a pathway to real-world decision making.	2
	10	No	None reported.	0
	11	No	None reported.	0
	12	No	None reported.	0
	13	No	None reported.	0
	14	No	None reported.	0
	15	Yes	Acknowledged in the acknowledgements section.	2
	16	Yes	The study was evaluated by participants in the fourth workshop and provided an overview of positive aspects and challenges of the workshops.	2
**Total Score = 21 (Medium-High)**				
	1	Yes	Objectives of the study were (1) delineate characteristics of built environment that are meaningfully different between summer and winter from the perspective of older adults; (2)ascertain which of the elements of the WHO age-friendly cities checklist	2

Garvin et al. (2012)			are relevant to Edmonton seniors and how; (3) identify and critically evaluate additional characteristics seniors identify as particularly helpful to consider in low-density, northern winter cities.	
	2	Unclear	Citizens are described as ‘participants’ but are engaged in photo-elicited focus group methods where they took photos of their environment a discussed these photos, which formed the data to be analysed and topics that are important to older adults. However, participants were reminded of project goal and purpose of research rather than being the drivers of this. The activities do engage participants in the methods, and they direct their photos and identify topics that are important to them. However, the project goals, purpose and data analysis and themes are all directed by the researchers so is a mix of citizen science and traditional.	1
	3	Yes	Citizens are clearly engaged in the photo-elicited methods in which they take their own photos, and inform discussions based on what is important to them. So citizens are engaged in the data collection stages but no other stages.	2
	4	Yes	The roles for participants are made clear in the focus groups, as well as through the photo-elicited approach	2
	5	Yes	It is clear that citizens are actively engaged in the photo-elicited approach and in the focus group discussions.	2
	6	No	None reported.	0
	7	Yes	The findings highlight the experiences and concerns of older adults in the summer and winter and provide pathways to real-world decision making in terms of the barriers and facilitators highlighted with photos.	2

	8	Unclear	Citizens were engaged as data collectors as well as identifying the key themes that are important. However, it is unclear how much the data-driven strategies and solutions were directed by citizens and how much were directed by researchers.	1
	9	Yes	The findings provide a pathway for designing and adapting urban environments based on the needs and experiences of older adults living in these spaces.	2
	10	No	None reported.	0
	11	No	None reported.	0
	12	No	None reported.	0
	13	No	None reported.	0
	14	No	None reported.	0
	15	No	None reported.	0
	16	No	None reported.	0
**Total Score = 14 (Medium)**				

Glover et al. (2020	1	Yes	This paper firstly provides a description of a theoretically in- formed co-creation study to investigate what it means to maintain health and well-being in older age and how to support this in a local context. Objectives were to: (a) develop a shared understanding of the meaning of healthy ageing, (b) identify barriers and facilitators to adopting behaviours that would support the identified essential components of healthy ageing, (c) make recommendations for adapting local ser- vices or developing new ones that are feasible, acceptable, and sustainable and (d) evaluate and make recommendations for authentic co-creation with older people.	2
	2	Yes	A co-creation approach was used in which citizens are actively involved in four group discussion meetings, with the theme of each meeting determined at the previous meeting (1st meeting theme decided by researchers). The study identifies itself as a 'co-create' study in which older adults are actively engaged.	2
	3	Yes	Citizens are clearly actively engaged in the discussion meetings as well as in evaluating the co-creation method. The group shared written and verbal information about the aims of the project and agreed both ground rules and levels of involvement	2
	4	Yes	Citizens are engaged and debate or share their views in each of the discussion group meetings as well as evaluating the project, so their roles are identified. The group shared written and verbal information about the aims of the project and agreed both ground rules and levels of involvement	2
	5	Yes	The extent to which citizens are engaged in clear in that they are steering the discussion group meetings and producing the recommendations.	2

	6	Unclear	The study does consider its limitation in terms of the location of the study, but it does not address data quality, bias, or limitations	1
	7	Yes	The findings of the study are clearly described and separated into relevant categories to address the objectives of the study.	2
	8	Unclear	The study argues that co-creation took place at every stage of the project from design to dissemination. However, it only reports that the data collection and recommendations are produced by older adults but do not report their engagement in driving the research focus, data analysis or the dissemination. Older adults were asked about the next steps in a questionnaire but the dissemination of this was not reported. The research question was already defined but it was argued that how it was addressed was shaped by older adults involved.	1
	9	Yes	The study presents outcomes and recommendations that could provide a pathway to real-world decision making, with feasible acceptable and sustainable recommendations for healthy ageing, as well as for recommendations for co-creation with older adults.	2
	10	No	None reported.	0
	11	Unclear	Citizens were asked after in a questionnaire about the key things that were discussed, what stood out and what should happen next. However, the outcomes of this were not clearly reported.	1

	12	No	Although the study says that co-creation was part of the study from data collection to dissemination, the dissemination part is not clearly reported at any stage.	0
	13	No	None reported.	0
	14	No	None reported.	0
	15	Yes	Acknowledged in the acknowledgements	2
	16	Yes	A process evaluation was completed based on group reflections through the co-creation experience and how this can be used/improved/ evaluated further as well as providing recommendations	2
**Total Score = 21 (Medium-High)**				
Gustafsson et al. 2018	1	Yes	the aim of this paper was to describe Life filming as a means of participatory approach among older community-dwelling persons regarding their local environment	2
	2	Yes	The study describes participatory methods and approaches, as well as empowerment of older adults through the participatory approach. This is through life filming which is a participatory approach and is used to collect data and capture older adults' experiences in which older adults describe their local environment and highlight areas in need of improvement using this	2

			approach. From planning to completion, the study describes continuous discussions of the purpose of the assignment, which strengthened groups awareness and commitment.	
	3	Yes	Citizens were part of a group there were given a goal to produce one film each about their local environment and attended information sessions and workshops to guide this (training of taking photographs and movies, homework assignments, filming of their environment and group discussions) which had a focus of learning and helping each other. Citizens also reflected on the process at each workshop to address their needs and perspectives. Representatives of the older persons who participated in the group activity also were involved in validating the findings collected by researchers. They were also part of guiding the dissemination of the videos they produced by demonstrating the need for them to be share with relevant municipal departments.	2
	4	Yes	It is clear that researchers are undertaking a descriptive single case study of the participatory methods, setting the community assignment and collect data through observations and memos. However, citizens are actively engaged in the different stages (see Q3), so the relationship is clearly reported.	2
	5	Yes	It is clear to what extent the citizens are actively engaged in the data collection through life filming and workshops (see Q3), alongside the authors collecting data from observations, conversations, and experiences from the group activity, and are part validating the data analysis. They are also part of the dissemination by demonstrating the need for them to be shared with	2

			relevant municipal departments and shared their videos with the city's website. This is a 'with the people' approach as there is a mixture of direction from the authors and citizens.	
	6	Yes	The authors consider the study design limitations and how the findings are based on a small number of participants and personal experiences which may limit transferability. They also highlight that the older adults were a group of 'healthy older people' with good socio-economic conditions which may have also affected the findings. They offer a solution for using a mixed-method approach or additional data sources to strengthen the study and to include a broader heterogenous group of older persons.	2
	7	Yes	The findings highlight Anchoring the concept of participation, Practical application of Life filming, The film as a product, Making a real difference, and An identity as a capable older person as the findings are clearly separated out.	2
	8	Unclear	Although the findings/solutions are based on outcomes from the active engagement of older adult citizens (in terms of the film as a product, the workshops which were based on learning and helping each other, and the dissemination of the films), part of this was directed by the researchers through the assignment given to the community centre, as well as from the data the authors collected through observations and field notes taken of older adults taking part in the group activity. It is unclear if the study outcomes area direct result of data-driven strategies generated by citizen scientists as instead, it is a mix of strategies driven by both citizens and researchers.	1

	9	Yes	The findings described a pathway to real world change in which the videos have been shared with the municipal government, shared at a national conference on city planning, and have been used for education purposes at Gothenburg University. The study also says ‘ any improvements in the local environment due to the action plan will also be reported’ showing that the videos are contributing and providing a pathway to real world change.	2
	10	Yes	The study intends to track the action plan for ‘age-friendly Gothenburg project’ when it is adopted and shared the outcomes from the videos that may contribute to local improvements with the citizens. Ripple effects were also highlighted in which older adults want to continue on after to teach others about how to produce films using tablets.	2
	11	Yes	Throughout the workshop, the study evaluated the citizens in terms of life filming to address their needs and perspectives and to include a reflective learning process throughout. The outcomes of this were highlighted in the findings through ‘an identify as a capable older person’ in which citizens wanted to learn about new technology and have continued on after to teach others about how to produce films using tablets.	2
	12	Yes	The study reports that it will continue to track and share the action plan for ‘age-friendly Gothenburg project’ when it is adopted and shared the outcomes from the videos that may contribute to local improvements with the citizens.	2
	13	No	Citizens are not mentioned in the publication process or asked to review the publication at any point	0

	14	No	The study is not in an open access format.	0
	15	Yes	Thanked in the acknowledgements	2
	16	Unclear	The citizens do take part in a reflective learning process throughout and highlight the positive aspects of life filming as an experience. However, there is no other critical evaluation of the study or its limitations	1
**Total Score = 26 (Medium-High)**				
Hand et al. (2018)	1	Yes	As such, this article describes an approach that draws on qualitative and geospatial methods aimed at understanding transactions between older adults and their neighbourhoods, illustrating its development and reflecting on its potential. The objectives are: To describe a combined qualitative-geospatial approach for studying of older adults in neighbourhoods; To investigate the qualitative-geospatial approach developed, including its utility and feasibility in exploring person–place transactions in neighbourhoods.	2
	2	Unclear	The study describes using CBPR approach and combining participatory geospatial and qualitative methods. The methods are described to capture in-depth story of older adults' experiences in their neighbourhoods, are grounded in their local environment and an advisory panel of older adult community members and other stakeholders were formed to contribute to decision making and collaboratively implementing the research approach. However, citizens are called 'participants' and took	1

			part in researcher directed combined methods of a narrative interview, a go-along interview and GPS tracking, completing an activity/travel diary and follow-up interview.	
	3	Yes	The methods chosen to do have active engagement of citizens in choosing the local destination and are engaged in informal interviews to discuss their walks, as well as discussing their maps after and to highlight their stories and provide meaningful data. However, they are part of semi-structured interviews with prompts, are tracked for 4 days using GPS and complete an activity diary and are not part of the data analysis of dissemination. This is a mixture of 'with' and 'for' the people with less active engagement.	2
	4	Unclear	The methods are set out clearly and the contribution of citizens is clear in that they are involved in each stage of the data collection which is directed by researchers. However, it is unclear if there is a partnership, or a shift from participant to active researcher.	1
	5	Unclear	Citizens participate in narrative interviews, go-along interview walks, tracked via GPS and discuss their maps. However, they are not engaged in the data analysis, and it is unclear if they are engaged in the dissemination of findings and results. Although citizens are provided with the opportunity to discuss their stories, It is unclear how much researchers directed the methods (i.e. study describes participants being 'observed' during their walks') and how actively engaged citizens are throughout these processes.	1

	6	No	The study does not discuss limitations or bias.	0
	7	Yes	The findings are clearly described and show the understandings gained through each stage of the combined methods used as well as highlighting reflections on the combined methods.	2
	8	Unclear	Although the methods provide an opportunity for citizens to engage and provide their stories, it is unclear if the findings and solutions are a direct result of data- driven strategies generated by the citizens or if they are driven by researchers.	1
	9	Unclear	The outcomes have impact for studies to further use combined methods to highlight how participants interact with their neighbourhood, highlights their experience and how they navigate their environments. It is unclear whether the findings have real world impact or could be taken to real-world decision making but instead would provide impact for further research on this topic that aim to use these methods.	1
	10	No	None reported.	0
	11	No	None reported.	0
	12	No	None reported.	0
	13	No	None reported.	0
	14	Yes	Open Access format	2

	15	No	None reported.	0
	16	Unclear	The study does provide a detailed overview of 'reflections on the combined methods' which highlights a positive evaluation only of the outcomes of the methods, however, does not provide an overview of its limitations or involve citizens in the evaluations.	1
**Total Score = 14 (Medium**				
Novek et al. (2014	1	Yes	The purpose of this study was to use a participatory methodology to explore older adults' perceptions of age-friendliness and to identify priorities and barriers to making communities more age-friendly.	2
	2	Yes	The study used a photovoice technique, described as a participatory research methodology) to address older adult's experiences within their social and physical environment. Older adults took photos to illustrate age-friendly features or barriers and recorded journal entries described each photograph, as well as taking part in interviews and discussions. Participants took photos and used a journal to describe the photos, were engaged in interviews after to elaborate further, and then attended group discussions to highlight priorities, give feedback on the themes. That had been generated and to identify how they wanted the findings to be used. An information session was held to explain the project and digital cameras, as well as info and instructions on how to use the camera. Participants were also given a journal to describe and interpret each photo they took. Participants were asked to select three priority photos which were used to compile a list of key issues for each	2

			community. Interviews were also undertaken after structured around the photos taken to elaborate on the photos. Participants also attended a focus group discussion to determine age-friendly priorities, which was based on the photos taken, and to give feedback on preliminary themes generated based on the photographs, journals & Interviews. At the end of the focus group, participants were asked how they wanted their findings to be used.	
	3	Yes	Participants are actively engaged in taking their own photos and describing them, elaborating further in interviews based on their photos, taking part in discussion groups to highlight priorities and give feedback on themes that had been produced, and highlighting how they want the findings to be used	2
	4	Yes	It is clear that participants are actively engaged in the data collection, and parts of the analysis and dissemination and are provided with an information session at the start to discuss the study.	2
	5	Yes	The study clearly highlights citizens are engaged throughout the data collection, in validating the themes produced and by identifying where and how they want their findings used.	2
	6	Yes	The limitation of the homogeneity of participants is considered and selection bias is discussed, with the suggestion of addressing perspectives of older adults from ethnic minorities in future research.	2
	7	Yes	Three categories of themes are presented clearly and show age-friendly features, contextual factors, and cross-cutting themes.	2

	8	Unclear	Although citizens are active data collectors, involved in the first stage of analysis, highlighting priorities within their data and identify where they want their information disseminated, it is unclear if they are active collaborators in terms of steering the project from start to finish. The study describes an information session which was used to 'explain the project' rather than to discuss the project and steer it. The majority of data analysis is completed by the researchers and a summary report was sent to participants and community members (service providers etc), but it is unclear if participants were involved in the development of the report aside from their photos and discussions.	1
	9	Yes	The study highlights key themes for 'age-friendliness', including physical environments, housing, business and services, activities, volunteering, social environment, affordability etc that could provide a pathway to real-world decision making for developing the urban environment for this group of older adults	2
	10	No	None reported.	0
	11	No	None reported.	0
	12	Yes	The study reports provide a summary report of age-friendly issues and recommendations which was shared with participants as well as service providers and community members.	2
	13	No	None reported.	0

	14	No	None reported.	0
	15	No	None reported.	0
	16	No	None reported.	0
**Total Score = 19 (Medium)**				
Parekh et al. 2018	1	Yes	The purpose of this study is to explore the role of social capital (e.g., social support through indirect ties) and social cohesion (e.g., interdependent support among neighbours) to unravel pathways for building age-friendly communities.	2
	2	Unclear	The study used a CBPR approach where three community liaisons assist in all phases of the study (planning, recruitment, data collection, translation & data analysis). Input from the community liaisons were integrated into the interview protocol and older adults were involved in member checking the final themes produced. However, the researchers collected data via face-to-face in-depth interviews with homebound older adults and focus groups for other older adults. A semi-structured interview guide used open-ended questions to address the experience of older adults. Aside from the community liaisons and member checking of themes, it is unclear if older adults were actively involved or collaborated in this study.	1
	3	Unclear	The methods chosen were used to understand 'aging well' from the 'lens of older adult participants', with the questions asked in interview elicit further responses and engaging individuals in conversations about what community means to them. The protocols also had input from community liaisons in older adult services. However, the degree of active engagement is	1

			unclear, as apart from member checking the final themes and engaging in conversations in interviews, they did not actively engage in other stages of the research.	
	4	Unclear	The roles and responsibilities are clear for the community liaisons, but it is unclear what the roles and responsibilities were for older adults apart from engaging in the interview/conversations and member checking the data analysis.	1
	5	Unclear	Citizens participate in the data collection stage by engaging in conversations and answering questions (although this is not active collaboration) and are also engaged in member checking the final stages of data analysis only but it is unclear if they are actively engaged any further or in the dissemination or use of data.	1
	6	Yes	The study considers the limitations in terms of the older adults recruited through community organisations, meaning those that do not interact with their community were not represented in the sample, as well as LGBTQ and other minority groups.	2
	7	Yes	The findings are clearly separated in clear sections for each theme and included quotes	2
	8	No	The study’s outcomes are not a direct result of data-driven strategies, although the strategies did have input from community liaisons, as the strategies and outcomes/solutions were guided by the researchers through interviews and data analysis.	0

	9	Yes	The findings provide pathway to real-world decision making for African American, Hispanic, and Vietnamese older adults in terms of the impact of their local community for participating in social cohesion & Civic Engagement, social inclusion barriers and the impacts of ageism.	2
	10	No	None reported.	0
	11	No	None reported.	0
	12	No	None reported.	0
	13	No	None reported.	0
	14	Yes	Open Access	2
	15	No	None reported.	0
	16	No	None reported.	0
**Total Score = 14 (Medium)**				
	1	Yes	The photovoice study reported in this paper was part of a wider study (doctoral) looking at approaches to evaluation of respect and social inclusion in older people in the context of an aspiring AFC. This study aimed to stimulate collective action	2

Ronzi et al. (2016			and advocacy to affect policy and empowerment by (i) encouraging dialogue between older people and city stakeholders at the exhibition; and (ii) by ensuring that older people's views were brought to the attention of city stakeholders so that they could include their concerns in decision making and planning processes for an AF	
	2	Yes	A CBPR method was used through photovoice and is described by the study to focus on co-creation knowledge, community empowerment and combines research with action. Participants participate in group discussion focus groups (which introduced the project and photography, received digital cameras, given a broad pre-identified problem and were asked to photograph this broadly as to limit the researcher influence over problems identified), took photos over a week (telling stories to highlight issues that are important to them), semi-structured interviews based on the photographs to select the most meaningful photos and the related stories (as well as a discussion on any photos that weren't taken), a second focus group where photographs were discussed, and researchers and participants agreed on the photos and descriptions for the exhibition. The findings are disseminated through a photo-exhibition to promote community discussions, policy and social change with participants, city stakeholders and members of the community.	2
	3	Yes	It is clear that citizens are actively engaged in each stage of the data collection (including taking the photos, telling the stories about the photos, discussing this with others and deciding on meaningful photos for the exhibition) and dissemination stage (taking part in the exhibit)	2

	4	Yes	An introductory focus group was undertaken to explain the project and to introduce citizens to the photography experience. It is clear that citizens are active researchers in the photos they take, described, and disseminated.	2
	5	Yes	Citizens are actively engaged in the data collection, and dissemination but are not engaged in the data analysis stage.	2
	6	Yes	The limitations of the citizen gender imbalance are considered as well as the limited time to take photos.	2
	7	Yes	The findings describe the opportunities, challenges, and solutions of using the photovoice methods and are separated into sections for the process to raise participants consciousness, factors influencing ability to take photos, negative aspects of photography, negative social concepts, time period for taking photos ,training, social expectations, and comfort using the camera, ethical aspects	2
	8	Unclear	Although citizens were engaged and empowered take photos on highlighting issues that are important to them as well as disseminating results at an art exhibition, and researchers tried to avoid influencing the photos taken, it is unclear in terms of citizen involvement in the structure and focus of the overall study, the interviews were semi-structured and data analysis were completed by researchers with no/unclear involvements of citizens.	1
	9	Unclear	Although the project provided pathway to real world decision making at the exhibition and with the report they provided, the findings reported in the study relate to the use of photovoice and the opportunities, challenges, and solutions for this. This would provide a good pathway for future research to use this method but unclear in terms of real-world decision making.	1

	10	No	None reported.	0
	11	Yes	In the findings, the study provided an overview of the photovoice methods experienced by participants including raising the participants consciousness (related to their city and awareness of respect/social inclusion etc) and thinking about what they want from the city.	2
	12	Unclear	The study reported a dissemination plan of providing a report with the findings to share with city stakeholders and follow-up this report and its impact, but it is unclear if citizens will be involved in this stage or if this report will be shared with them.	1
	13	No	None reported.	0
	14	Yes	Open Access.	2
	15	Yes	Thanked in acknowledgements	2
	16	Yes	At the end of the focus group, participants were asked how they experienced the photovoice project and how it could be improved in the future. The exhibition event was also assessed with a short evaluation survey.	2
**Total Score = 25 (Medium-High)**				

Tuckett et al. (2018)	1	Yes	1)What are the features that help or hinder access to a seniors' center? 2)What are the features of the physical environment surrounding a seniors' center that help or hinder physical activity (walking)?; 3)In what ways can older adults acting as citizen scientists bring about changes to their local environment?	2
	2	Yes	A Citizen Science framework was used (our voice) for collaborative research where citizens undertook a walk using a digital tool to take photos, audio narratives and GPS track the walks. Citizens were also engaged in the data analysis and the dissemination of the findings through activate sessions through public presentations.	2
	3	Yes	Yes it is clear that citizens are actively engaged in data collection (the walk to take digital photos, audio narratives and GPS track the walks and individually designated a feature as helpful or hindrance), Photos were printed and preliminary content coded elements were derived from the walks (by researchers) for the discussion group, data analysis(Citizens took part in a further discussion group phases where they coded their own data (facilitated by researchers) by assigning photos graphs with paired audio transcripts under the code element headings to validate the codes. Once this was completed, the citizens reached a consensus on the data and two citizens were nominated to advocate the groups findings and solutions at an 'activate' session. A collective brainstorming of solutions and priorities also took place with citizens which were recorded on a photograph board) and disseminating the outcomes (two activate sessions were completed by the two citizen scientists (driven by citizens, with the second session co-delivered with researcher through public presentations to municipal council members and to the public to discuss group needs.)	2

	4	Yes	A community partnership process is undertaken where citizens are actively engaged as co-researchers in the research and the roles/expectations are clear to citizens. and are involved in steering the data collection, data analysis and dissemination of data, which is clear.	2
	5	Yes	Yes, this is a by the people approach in which citizen scientists are co-research and are involved in steering the data collection, data analysis and dissemination of data	2
	6	Yes	Research limitations were considered in terms of small sample size in which the outcomes need to be weighed carefully against sample size (Outcomes need to be weighed carefully against sample size/ caution must be taken about extrapolating outcomes), predominantly female sample, and reasonably short time frames in which impacts of the intervention have been evaluated.	2
	7	Yes	The findings are broken down into examples of the coded elements with relevant photos taken (i.e. footpaths, playgrounds)	2
	8	Yes	Citizens were actively engaged in steering the data collection, and actively collaborating in the discussion groups, highlighting action points and solutions, and advocating and disseminating the results.	2
	9	Yes	The findings provide coded elements of the physical environment with photos that could provide a pathway to real world decision making through adapting and changing the physical element. The advocating of the results also led to ‘the Councillor committed to making the citizen scientists’ proposed changes.	2

	10	Unclear	The study does report ripple effects from the project in terms of the work that is being planned or has taken place (line marking road works, footpath repairs), approval for construction of a new toilet block. However it does not report long term impacts or changes (or intention to track) for citizens.	1
	11	No	None reported.	0
	12	No	None reported.	0
	13	No	None reported.	0
	14	Yes	Open Access	2
	15	Yes	None reported.	2
	16	No	None reported.	0
**Total Score = 24 (Medium-High)**				
Verma & Huttunen (2015)	1	Yes	The aim of this case study was to gain understanding of the user experience of the living environment integral to further local development.	2
	2	Unclear	Participatory user study methods are described (workshops and walk-throughs, together with an internet-based online questionnaire were used) with workshops focused on daily paths and current/need for services were the focus and pictures of	1

			the local environment and a map were used to facilitate the workshop where older adults shared their experiences and impressions of the living environment/ services they would like/dislike in their neighbourhood. Three walk-through assessments were completed to evaluate users personal experience through discussion and observation, as well as routes, walking speeds and resting spots collecting on a mobile map application. The findings from this walk-through helped to formulate the online questionnaire about individual's local environment. It is unclear if this is a citizen science approach as although they are described as participatory user study methods, citizens are not involved in the planning or focus of the study, the analysis, dissemination and during the data collection they only provide data in terms of their experience and insights, but the photos/routes are already chosen.	
	3	Unclear	The methods chosen provide active engagement in terms of older adults providing individual and collective experiences, discussing things they want or dislike in their neighbourhood and reporting their experiences through the tour. However, the active engagement is unclear as the workshops were guided by the researchers (they chose the route from homes to the library, provided the photos, and it is unclear how the discussion was facilitated) and the walks were organised by organisations or the researchers and called a 'tour' where older adults provided their observations during the walk. Older adults were also not engaged in the planning of the study and data analysis is not mentioned.	1
	4	Unclear	The roles and responsibilities are clear between citizens and researchers, but it is unclear if citizens are more participants who provide data rather than active researchers (i.e. they were provided pictures, walks were organised by association or	1

			researchers rather than citizen, they were not involved in the data analysis or the planning of the research) and it is unclear if there is a clear partnership between researchers and citizens.	
	5	Unclear	Citizens are actively engaged in the data collection to share their in-depth knowledge, experiences, and impressions but they are not actively engaged in the data analysis or dissemination. The active engagement in data collection is unclear as the workshops were guided by the researchers (they chose the route from homes to the library, provided the photos, and it is unclear how the discussion was facilitated) and the walks were organised by organisations or the researchers and called a 'tour' where older adults provided their observations during the walk. Older adults were also not engaged in the planning of the study and data analysis is not mentioned.	1
	6	Unclear	The study does consider its limitations briefly - 'This study has limitations, as this research was conducted in a particular place over a certain period of time. The participants represented 2% of the elderly living in the area.' but does not discuss data limitations or biases further or how these limitations could be addressed.	1
	7	Yes	The main findings are separated into features of the environment and the experience or impressions from older adults about these features.	2
	8	No	Citizens are engaged in the data collection to provide their insights and experiences but are not active collaborators in terms of the data-driven strategies and solutions produced.	0

	9	Yes	The findings provide a pathway to real world decision making in terms of identifying the local services and features (green spaces, transportation etc) that are important to older adults in this area and can be adapted, changed, or promoted.	2
	10	No	None reported.	0
	11	No	None reported.	0
	12	No	None reported.	0
	13	No	None reported.	0
	14	No	Not Open Access.	0
	15	No	None reported.	0
	16	No	None reported.	0
**Total Score = 11 (Medium)**				
	1	Yes	The main aim of this paper is to describe how participatory video design can add knowledge about the preferences and needs of older people about the improvement and preservation of their local environment.	2

Von Faber et al. (2020)	2	Yes	We focus on participation in research, enabling older people to seek their own solutions according to their priorities. A participatory video method is used as co-creative approach.	2
	3	Yes	Representatives and a regional advisory board of older people helped shape the focus of the study, the neighbourhoods where participants were recruited and took part alongside other older adults in the data collection. Citizens (both the representatives and the older adults recruited) actively engaged in a 5-day workshop to produce a film and disseminate it to family and friends based on a topic.	2
	4	Yes	It is clear that there is a partnership between researchers and the regional advisory board who are there to provide insight and steering to the project focus. Citizens also take part in the study to develop, film and disseminate a film on a specific topic.	2
	5	Yes	Citizens are engaged in developing, filming and disseminating the film on a specific topic. They write the script, chose the aspects to film, edit their films and disseminated it to family members	2
	6	Yes	The study considers a potential limitation of visual methods in that it could exclude persons with impairments related to vision, hearing, or mobility. This could introduce a selection bias and under-representation of vulnerable groups.	2
	7	Yes	The findings are broken down based on the topics of the films and clearly identify the barriers and facilitators of cities that influence older adults. The process of participation and ways to improve the film making process are also documented in the results.	2

	8	Yes	The findings are those that are directly produced by older adults who developed their own subtopics of the films, such as outdoor spaces, social participation and characteristics of the neighbourhoods. Citizens were actively engaged in writing their own scripts and going out and filming their own films which produced the topics for the findings.	2
	9	Yes	The findings demonstrated the preferences and needs of the older adults in these locations and demonstrated features and characteristics that could be used to develop age-friendly spaces. This produces a pathway to making real-world decision-making based on these characteristics.	2
	10	No	Although the study did provide the opportunity for a follow-up activity by participating in a focus group to discuss the final results and further suggestions for improvements and reported the neighbourhood association was also reported to take responsibility of improving mobility and safety, there was no intention to track these changes and their impacts further, alongside any further participatory activities of citizens. The study also reported that no arrangements were made to evaluate and discuss the results of the video research.	0
	11	No	None reported	0
	12	Unclear	The study reports sharing the film with citizens who wanted to receive their film. However, no other dissemination is reported.	1
	13	No	None reported.	0

	14	Yes	Open access article.	2
	15	Yes	Acknowledged in the acknowledged sections	2
	16	Yes	An evaluation was completed by participant experience during the film making processes and provided an overview of the experience, and suggestions for improvement such as more time to complete filming.	2
**Total Score = 25 (Medium-High)**				
Ronzi et al. (2020)	1	Yes	We employed a Photovoice methodology within a Community-Based Participatory Research approach to: (i) explore the extent to which respect and social inclusion were promoted as the city sought to become more age-friendly/an AFC, and (ii) actively involve older adults in the research process and to allow them to directly communicate issues with stakeholders involved in AFC policy.	2
	2	Yes	A photovoice methodology within a CBPR approach was employed to involve older adults in identifying priorities for action and in decision0making processes. It was used to provide a unique perspective on the issue of respect and social inclusion in the urban context whilst creatively involvement participants in the research process.	2
	3	Yes	The active engagement of citizens is presented in six phases were completed which included: 1) Discussion group providing an overview of the project aims, a focus group discussion to explore perceptions of research and social inclusion, photographical and ethical trained, and were asked to photograph aspects of their environment that enabled or prevented	2

			feeling valued and part of the community and to identify potential solutions; 2) Participants spent a week taking photos; 3) Each participant selected six photographs they wanted to discuss during an in-depth semi-structured interview and focus group; 4) A second focus group discussion took place where photographs were collectively interpreted and key themes emerged from the discussions. Participants also discussed how they wanted to communicate their findings to policy makers and relevant stakeholders; 5) Captions were developed for participant chosen photos by researchers and reviewed by participants for the photo-exhibition; 6) Photos were shown at a public and stakeholder event where participants presented their photos and communicate directly to stakeholders. Data analysis was completed by the researchers without member checking.	
	4	Yes	It is clear that citizens are engaged to actively collect data through photovoice, identify key photos and solutions, and disseminate them with community stakeholders. Researchers are present to facilitate the process (i.e. providing an overview of the project aims, facilitating ethical training and complete data analysis).	2
	5	Yes	Citizens are engaged in actively collecting and disseminating the data. However, they are not engaged in the data analysis stage where researchers completed thematic analysis.	2
	6	Yes	The limitation related to the gender imbalance in the sample was considered (males: 7; females: 19). The guiding principle for recruiting participants was to have a mix of included and less included participants, rather than focusing on gender differences. Moreover, we realised that (i) our strategy to recruit participants from grassroots organisations in order to include	2

			a mix of more or less socially included older adults and (ii) our requirement for participants to physically attend the sessions and being able to walk (even if for short distances only) to take photographs, meant that we did not reach some of the most excluded older people.	
	7	Yes	The outcomes are presented in relation to the urban context that fostered or hindered respect and inclusion, which included the physical environment, transportation, public facilities, disrespectful environmental attitudes, social environment, places to cultivate informal and formal relationships and negative age perceptions, disrespectful attitudes and neighbourhood fragmentation.	2
	8	Yes	The outcomes are based on the photos taken from the active data collection completed by citizens through the photovoice and focus group discussions. Citizens were also engaged to advocate their photos with community stakeholders.	2
	9	Yes	The outcomes provide an overview of the contexts and features that hinder or facilitate respect and social inclusion for older adults in Liverpool, and these are aspects that can provide a pathway for being developed or altered to facilitate this further	2
	10	No	None reported	0
	11	No	None reported	0
	12	No	None reported	0

	13	No	None reported	0
	14	Yes	Open Access	2
	15	Yes	Citizens acknowledged in the acknowledgements	2
	16	No	None reported	0
**Total Score = 22 (Medium-High)**				
Salma et al. (2020)	1	Yes	This study elicits perceptions of aging and related needs of immigrant Muslim communities in an urban center in Alberta by answering two questions: What are immigrant Muslim older adults' experiences of growing old in their communities? ; What are the stakeholders' perceptions of the needs for aging well in immigrant Muslim communities?	2
	2	Yes	The study discusses community-based participatory principles that are used for 'power-equalizing' and follows principles of shared leadership, collaborative decision-making and researcher-community trust-building	2
	3	Yes	. A community advisory group of stakeholders and older adults participated in all stages of the research process via monthly meetings and drafting the interview guide, identifying stakeholders, recruiting older adults and informing data analysis. Older adults themselves were only actively engaged in the interview or focus group process. This demonstrates a 'by the people' due to the advisory group but a 'with the people' due to the only active engagement of other older citizens in the interview/focus group process.	2

	4	Yes	A relationship has been built with the lead research and the advisory group/ older adults engaging in the study and the roles are clear about their level of engagement and expectations.	2
	5	Yes	Older citizens are actively engaged in the data collection stage only, and the advisory group are engaged in informing the last step of data analysis. No involvement of older adults or advisory group is discussed for dissemination.	2
	6	Yes	The study considers their limitations through the following: 1)The majority of participants were low-income women belonging to Arab, South Asian, or African immigrant communities. The findings of this study are not necessarily transferable to other immigrant Muslim groups; 2) The study took place in only one province and city in Canada. The traditional immigrant-receiving locales of Vancouver, Montreal, and Toronto have higher percentages of immigrant older adults, which might interpret into more resources available to this population and different aging experiences.	2
	7	Yes	The findings are reported in three clear categories: 1) Aging while living across place; 2) Negotiating access to aging-supportive resources in a time of scarcity; 3) Re-envisioning Islamic approaches to eldercare.	2
	8	Unclear	Although an advisory group were engaged in the planning of the interview guide, the recruitment, and the data analysis, and older adults were actively engaged in focus groups, the researchers directed the choice of methods, the data collection and it is unclear if citizens were fully engaged as active collaborators.	1

	9	Yes	The outcomes identify the needs of older adult Muslim's residing in Canada and can be used as a pathway to real-world decision making i.e. the need for religious, cultural and spiritual considerations to be incorporated into eldercare.	2
	10	No	None reported	0
	11	No	None reported	0
	12	No	None reported	0
	13	No	None reported	0
	14	No	Not open access	0
	15	Yes	The community members are thanked in the acknowledgements section	2
	16	No	None reported	0
**Total Score = 19 (Medium)**				

2. CSAT Medium and Low Quality Studies

2.1 CSAT Medium Quality

Studies in this category (n=8) scored highly in parts of the first three sections of the tool (Figure 6). All studies provided a clear description of aims, objectives and main findings. Six studies (75%) presented the degree of active engagement of citizens and the roles and type of partnership between citizens and researchers[1-6] considered their data limitations or biases,[1-3, 6-8] and demonstrated 'real world' implications or pathways for their outcomes.[1-4, 6, 8]

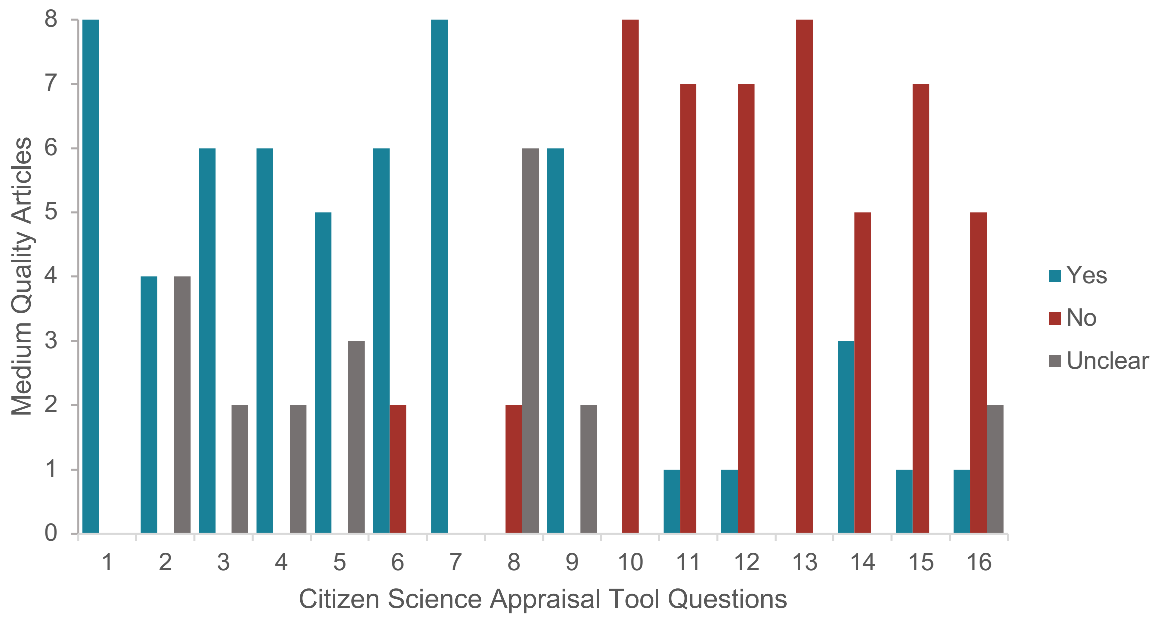

Fig I Citizen Science Checklist Tool Outcomes for Medium Quality Articles

Studies in this category could be further strengthened in the outcomes and knowledge section by providing intention to track long-term or 'ripple effects', acknowledging citizens in the publication, and inviting citizens to review or participate in the publication process. Studies

could also provide accessible dissemination plans or mechanisms for sharing outcomes and provide an evaluation of citizen knowledge or behaviour, and methods or limitations.

The level of citizen science engagement varied, with 6 studies demonstrating ‘by the people’ and ‘with the people’, [2-6, 8] and 2 demonstrating ‘with the people’ only. [1, 7] Citizens were actively engaged in the data collection in all 7 studies, with those demonstrating a ‘by the people’ approach engaging citizens to member check data,[2-5] and take part as community liaisons [3, 8] or advisory group.[5, 6] These studies could strengthen their CS approaches by actively engaging citizens further in the planning and design, directing of the research methods, and taking part in data analysis and dissemination of outcomes.

2.2 Low-Medium Quality

One study [9] provided a clear statement of its aims and outcomes, and its findings showed a pathway to ‘real world’ decision making implications. This study could be strengthened in all sections of the tool by providing a clear overview of the citizen science or participatory approach used and facilitate further the degree of active engagement of citizens. The study could also provide tracking of long-term impacts or ripple effects, an evaluation of citizen knowledge or behaviours and methods or limitations, acknowledge citizens in the publication, and provide accessible dissemination plans for sharing outcomes.

A ‘with the people’ engagement was present in this study as older adults were actively engaged in the data collection processes by sharing their experiences or views. However, researchers also collected by part of the data through observations and did not engage citizens in any other stages of the research process. Although this study defined its participatory approach as user methods, it could strengthen this approach by actively engaging citizens further throughout the entire research process.

The role of urban environments in promoting active and healthy ageing: A systematic scoping review of citizen science approaches Supplementary Material 2

References

1. Aw S, Koh G, Oh YJ, et al. Explaining the continuum of social participation among older adults in Singapore: From 'closed doors' to active ageing in multi-ethnic community settings. Empirical Study; Interview; Focus Group; Qualitative Study. *Journal of Aging Studies*. Aug 2017;42:46-55. doi:http://dx.doi.org/10.1016/j.jaging.2017.07.002

2. Novek S, Menec VH. Older adults' perceptions of age-friendly communities in Canada: a photovoice study. *Ageing & Society*. 2014;34(6):1052-1072. doi:10.1017/S0144686X1200150X

3. Adorno G, Fields N, Cronley C, Parekh R, Magruder K. Ageing in a low-density urban city: transportation mobility as a social equity issue. *Ageing & Society*. 2018;38(2):296-320. doi:10.1017/S0144686X16000994

4. Garvin T, Nykiforuk CIJ, Johnson S. Can we get old here? Seniors' perceptions of seasonal constraints of neighbourhood built environments in a northern, winter city. Article. *Geografiska Annaler, Series B: Human Geography*. 2012;94(4):369-389. doi:10.1111/geob.12004

5. Hand CL, Rudman DL, Huot S, Gilliland JA, Pack RL. Toward Understanding Person--Place Transactions in Neighborhoods: A Qualitative-Participatory Geospatial Approach. *Gerontologist*. 2018;58(1):89-100. doi:10.1093/geront/gnx064

6. Salma J, Salami B. “We Are Like Any Other People, but We Don’t Cry Much Because Nobody Listens”: The Need to Strengthen Aging Policies and Service Provision for Minorities in Canada. *The Gerontologist*. 2020;60(2):279-290. doi:10.1093/geront/gnz184

7. Chui CH, Chan OF, Tang JYM, Lum TYS. Fostering Civic Awareness and Participation Among Older Adults in Hong Kong: An Empowerment-Based Participatory Photo-Voice Training Model. *J Appl Gerontol*. Mar 21 2019:733464819838448. doi:10.1177/0733464819838448

8. Parekh R, Maleku A, Fields N, Adorno G, Schuman D, Felderhoff B. Pathways to age-friendly communities in diverse urban neighborhoods: Do social capital and social cohesion matter? *Journal of gerontological social work*. 01 Jul 2018;61(5):492-512. doi:http://dx.doi.org/10.1080/01634372.2018.1454564

9. Verma I, Huttunen H. Elderly-friendly neighborhoods. *Journal of Housing for the Elderly*. 2015;29:1-2.
